# M-band wavelet-based multi-view clustering of cells

**DOI:** 10.1371/journal.pcbi.1013060

**Published:** 2025-05-23

**Authors:** Tong Liu, Zihuan Liu, Wenke Sun, Adeethyia Shankar, Yongzhong Zhao, Xiaodi Wang

**Affiliations:** 1 Department of Mathematical Sciences, Tsinghua University, Beijing, China; 2 Data and Statistical Science, AbbVie, Chicago, Illinois, United States of America; 3 School of Economics and Management, Dalian University of Technology, Dalian, China; 4 Brown University, Providence, Rhode Island, United States of America; 5 Frontage Labs, Exton, Pennsylvania, United States of America; 6 Department of Mathematics, Western Connecticut State University, Danbury, Connecticut, United States of America; University of Maryland School of Medicine, UNITED STATES OF AMERICA

## Abstract

Wavelet analysis has been recognized as a widely used and promising tool in the fields of signal processing and data analysis. However, the application of wavelet-based method in single-cell RNA sequencing (scRNA-seq) data is little known. Here, we present M-band wavelet-based scRNA-seq multi-view clustering of cells (WMC). We applied for integration of M-band wavelet analysis and uniform manifold approximation and projection (UMAP) to a panel of single cell sequencing datasets by breaking up the data matrix into an approximation or low resolution component and *M*–1 detail or high resolution components. Our method is armed with multi-view clustering of cell types, identity, and functional states, enabling missing cell types visualization and new cell types discovery. Distinct to standard scRNA-seq workflow, our wavelet-based approach is a new addition to uncover rare cell types with a fine resolution.

## Author summary

We develop M-band wavelet-based multi-view clustering method of cells. Our new approach integrates M-band wavelet analysis and UMAP to a panel of single cell sequencing datasets via breaking up the data matrix into an approximation or low resolution component and *M*–1 detail or high resolution components. Our method enables us to examine multi-view clustering of cell types, identity, and functional states, potentializing missing cell types recovery, rare cell types discovery, as well as functional cell states exploration.

## Introduction

Recent breakthroughs in methodology are enabling the study of the transcriptomes of individual cells, which paves the way for more objective investigations of cellular functions at single-cell level [1–3]. A primary objective of single-cell RNA sequencing (scRNA-seq) analysis is to identify and discover cell types [4–7], identities [8], states [6, 9], as well as accompanied gene signatures [10, 11], while conventional pipelines have been unsatisfying due to limited resolution even with missed critical cell types, identity, and functional states. Earlier studies based on microscopy [12], histology [13], and pathological criteria [14] have contributed to the resolution of this problem. Recent efforts have utilized both unsupervised and supervised clustering techniques to cluster cells alongside transcription signatures [5, 7] yet with big room to be optimized, requesting novel approaches.

Those limitations in the existing methods have motivated us to develop a new method, aiming to recover missing cell clusters and uncover rare cell types. Wavelet frameworks have been successfully applied to numerous tasks in the biomedical domain, such as genomics [15], promoters [16, 17] and GWAS [18]. However, wavelet-based method in scRNA-seq data analysis has been largely unexplored.

Our hybrid technique incorporated with M-band orthogonal wavelet enables us to illustrate multi-view clustering of cell types, identities, and functional states from a variety of perspectives simultaneously. Distinct from the regular RNA-Seq analysis pipelines, our strategy have the power to uncover missed and rare cell types, identities, states, via wavelet-based multi-view of clustering (WMC). By taking a weighted-average like process on the original RNA-Seq matrix, The trend component of the M-band wavelet enables us to take an approximation of the original data, while the detail components provides the detailed information.

## Results

### The overview of WMC

We aim to build a pipeline with multi-view of clustering on scRNA-seq data. Stemming from the original scRNA-seq workflow, our WMC integrates wavelet-based multi-view clustering into standard pipelines. In the light of wavelet analysis, we develop WMC to present multi-views of scRNA-seq in different resolution windows, potentializing recovery of missing cell types, cell states, cell identities, as well as rare cell types discovery. Our WMC consists of the following steps, including quality control, log-transformation, discrete wavelet transform (DWT), dimension reduction, multi-view clustering visualization, along with an assessment of intersecting components.

**Fig 1:**
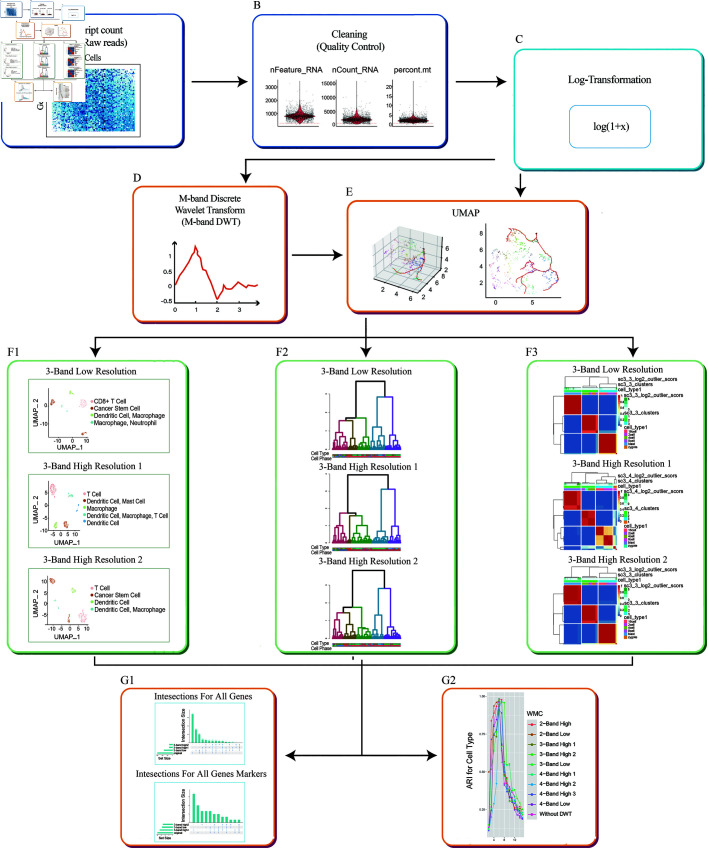
Illustration of the WMC workflow. ( **A**) Raw[0pc][-1pc]Figure 1,3,4 - The quality of the image is poor and pixelated. Hence please supply a corrected version with an unpixelated typeface. The font size of the image is below 6 pt which affects the readability of the image. Hence, please supply a corrected version with font size above 6 pt. scRNA-seq data matrix with row as transcripts and columns as individual cells. ( **B**) Quality control diagrams, demonstrating the process of removing unqualified cells and transcripts involving mitochondrial genes. ( **C**) Logarithm transformation of the raw matrix. ( **D**) Applying M-band DWT on the logarithm transformed matrix. ( **E**) PCA-based dimension reduction on matrices with and without DWT. ( **F**) Visualization of clustering based on different methods, with (F1) for UMAP in Seurat, (F2) for SC3 and (F3) for HGC. ( **G**) Assessing the performance of WMC via intersection analysis and ARI computation.

Given a raw scRNA-seq data matrix (Fig 1A), we remove the low-quality cells (Fig 1B) to ensure that technical effect does not distort downstream analysis results. Next, we apply a logarithmic transformation to the raw data matrix (Fig 1C), followed by M-band DWT, which decomposes the normalized data matrix into an approximation component and *M*–1 detail components (Fig 1D).This decomposition enables the extraction of multi-scale components, facilitating multi-view clustering of cell types, identities, and functional states from multiple perspectives. To further reduce dimensionality, we apply uniform manifold approximation and projection (UMAP) to both wavelet-transformed and non-transformed matrices for exploratory visualization (Fig 1F). Clustering is performed using the default method in Seurat [19]. In particular, on PCA-reduced matrix, we implement Louvain community detection algorithms [20] on single-cell K-Nearest Neighbor (KNN) graph to organize cells into clusters. To characterize the similarities and differences between the classic and our proposed method, we compare the clusters based on both original and wavelet-transformed multi-view matrices (Fig 1G). Of note, we use the single-cell Cluster-based automatic Annotation Toolkit for Cellular Heterogeneity (scCATCH) [21] for cluster annotation. In addition to the Seurat pipeline, we incorporate alternative clustering methods, including Single-Cell Consensus Clustering (SC3) and Hierarchical Graph-based Clustering (HGC), to wavelet-transformed matrices, demonstrating the compatibility of WMC with multiple clustering frameworks. To assess the performance of WMC multi-view clustering, we evaluate the overlap of marker genes identified by WMC and conventional methods without DWT. Additionally, the adjusted rand index (ARI) and normalized mutual information (NMI) are computed, resulting in similar cluster consistency quality across distinct approaches. Yet WMC enables more clusters revealed. Thus, WMC has a multi-view function without compromise of cluster quality.

### Mathematical statement of WMC

Let $S=[s_{1},...,s_{p}]\in {\mathbb{R}}^{n\times p}$ denotes the original data matrix and $W=[w_{1}$,...,$w_{n}{]}^{T}\in {\mathbb{R}}^{n\times n}$ be the corresponding M-Band DWT matrix [22, 23]. Then, the M-Band DWT of $s_{i}$ for i=1,...,p is given by

$$Ws_{i}=[a(i),d_{1}(i),d_{2}(i),...,d_{M-1}(i){]}^{T}\;$$ (1)

where *n* = *Mk*, $a(i)=[a_{i1},a_{i2},...,a_{ik}{]}^{T}$ and $d_{j}(i)=[d_{j,i,1},d_{j,i,2},...,d_{j,i,k}{]}^{T}$ for j=1,2,...,M−1. For brevity, we denote $Ws_{i}$ by ${\tilde{s}}_{i}$. Let $\tilde{S}=[{\tilde{s}}_{1},{\tilde{s}}_{2},...,{\tilde{s}}_{p}]$, then

$$WS=\tilde{S}=[a,d_{1},...,d_{M-1}{]}^{T}$$ (2)

where a=[a(1),...a(p)] being the low resolution component (or trend) of *S*, and $d_{1}=[d_{1}(1),...,d_{1}(p)]$, ... ,$d_{M-1}=[d_{M-1}(1),...,d_{M-1}(p)]$ being the high resolution components (or fluctuations) of *S* in wavelet domain. Since *W* is an orthogonal matrix, $\{w_{1}$,$w_{2}$,...,$w_{n}\}$ forms an orthonormal basis of ${\mathbb{R}}^{n}$. The components of ${\tilde{s}}_{i}$ are also called the wavelet coefficients of $s_{i}$. Therefore, the components of ${\tilde{s}}_{i}$ are coordinates of $s_{i}$ under this wavelet basis. Due to the orthonormality of *W*, for i=1,2,...,p, we have $\left||{\tilde{s}}_{i}||=||s_{i}|\right|$ and $W^{T}=W^{-1}$. Hence the M-Band DWT preserve the length or energy of vectors it transforms. If we multiply both sides of (2) by $W^{T}$, we obtain

$$S=W^{T}\tilde{S}=A+D_{1}+...+D_{M-1}$$ (3)

and

$$||s_{i}|{|}^{2}=||A_{i}|{|}^{2}+||D_{1,i}|{|}^{2}+...+||D_{M-1,i}|{|}^{2}\;=||a(i)|{|}^{2}+||d_{1}(i)|{|}^{2}+...+||d_{M-1}(i)|{|}^{2},$$ (4)

where

$$A=[A_{1},A_{2},...,A_{P}]=[w_{1},w_{2},...,w_{k}]a,\;D_{j}=[D_{j1},D_{j2},...,D_{jp}]=[w_{kj+1},w_{kj+2},...,w_{kj+k}]d_{j},j=1,2,...,M-1.$$ (5)

In other words, we use M-band DWT to decompose *S* into the sum of M orthogonal components (multi-view windows), where one low-resolution component represents the approximation part, while the remaining *M*–1 components, which are of high resolution, correspond to the detail parts, as shown in (3)

We next aim to find *A*_*i*_ and *D*_*j*_ for i=1,...p and j=1,2,...,M−1. Define $𝒱=span\{w_{1},w_{2},...,w_{k}\}$, ${𝒲}_{i}=span\{w_{ki+1},w_{ki+2},...,w_{ki+k}\}$, i=1,2,...,M−1, then 𝒱,${𝒲}_{1},...,$ and ${𝒲}_{M-1}$ are orthogonal subspaces of ${\mathbb{R}}^{n}$. It follows that,

$$𝒱⊕{𝒲}_{1}⊕...⊕{𝒲}_{M-1}={\mathbb{R}}^{n}$$ (6)

Let ${𝒫}_{Y}x$ be a projection of x on *Y*, we have

$$A_{i}={𝒫}_{V}s_{i}\;D_{j}={𝒫}_{W_{j}}s_{i}$$ (7)

We then apply different clustering methods on *S* and its corresponding components $A,D_{1},...,D_{M-1}$ to obtain their multiview images as ℐ(S), $ℐ(A_{1})$, $ℐ(D_{1})$,..., $ℐ(D_{M-1})$, resulting in multiview clusters with principal cell names, encompassing both approximation and detail parts of *S*. Thus, the essemble of annotated gene clusters enables us a multi-views cell types, identities, and states with sc-RNA seq data.

We assess the performance of WMC via computing the intersection of ℐ(S), $ℐ(A_{1})$, $ℐ(D_{1})$,..., $ℐ(D_{M-1})$. Since the space of different components are orthogonal to each other, few genes could be found in the intersection of different components theoretically, Therefore, the smaller number of genes appearing in the intersection of these components yields a better resolution of clustering.

### Cluster analysis of the scRNA-Seq data set by Wavelet-Seurat routine

We apply Wavelet-Seurat to a benchmark scRNA-seq dataset profiling 2,613 peripheral blood mononuclear cells (PBMCs) from 10 × Genomics. Specifically, WMC identifies 8, 10, and 13 cell types under 2-band, 3-band, and 4-band UMAP settings, respectively, whereas only six cell types are identified using the standard method without DWT (Fig A in S1 Text). In addition to the six cell types which have been identified by the regular method, WMC discovers novel clusters, including natural killer (NK) cells with 2-band and 3-band DWT, non-switched memory B cells with 3-band and 4-band DWT, as well as regulatory T cells, memory T cells, and SLC16A7+ cells with 4-band DWT. Furthermore, additional unknown cell types are identified from the detailed components in 3-band and 4-band DWT.

To further evaluate our Wavelet-Seurat approach, we have analyzed a published scRNA-seq dataset from a colorectal cancer patient. A similar trend is observed, as Fig B in S1 Text illustrates that the standard UMAP setting identifies six cell types, while 2-band, 3-band, and 4-band UMAP settings reveal 9, 12, and 12 cell types, respectively. Beyond the cell clusters observed in both the conventional and WMC methods, our approach identifies additional clusters, including mitotic fetal germ cells, astrocytes, monocytes, fibroblasts, plasma cells, and unknown cell types.

**Fig 2:**
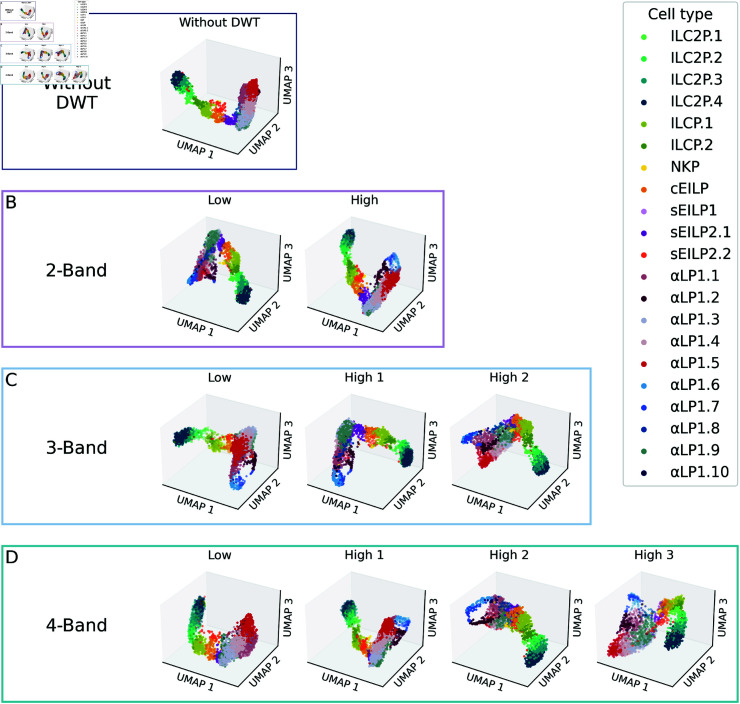
Multi-view of clusters of the innate lymphoid cell differentiation dataset. ( **A**) UMAP visualization of cell types based on data matrix without DWT. ( **B**)-( **D**) are clusters under wavelet analysis, with ( **B**) for 2-band DWT, ( **C**) for 3-band DWT, and ( **D**) for 4-band DWT.

Moreover, we visualize the cell-type representations learned by Wavelet-Seurat in a three-dimensional UMAP space using the innate lymphoid cell (ILC) differentiation dataset (Figs 2 and G in S1 Text) [24]. After quality control, this dataset contains 2,544 cells spanning three developmental phases. Using the standard Seurat framework, we identify clusters corresponding to ILC2P, ILCP, NKP, cEILP, and sEILP families, along with six types of alpha-LP cells. However, applying wavelet transform further resolves alpha-LP cells into 10 distinct subtypes, revealing additional clusters such as alphaLP1.7, alphaLP1.8, alphaLP1.9, and alphaLP1.10, resulting in a total of 21 distinct cell clusters. Overall, wavelet transformation provides a multi-view perspective by projecting the dataset into different component spaces, enhancing the resolution of cell-type identification and enabling finer-grained clustering.

### Cluster analysis using Wavelet-SC3 routine

**Fig 3:**
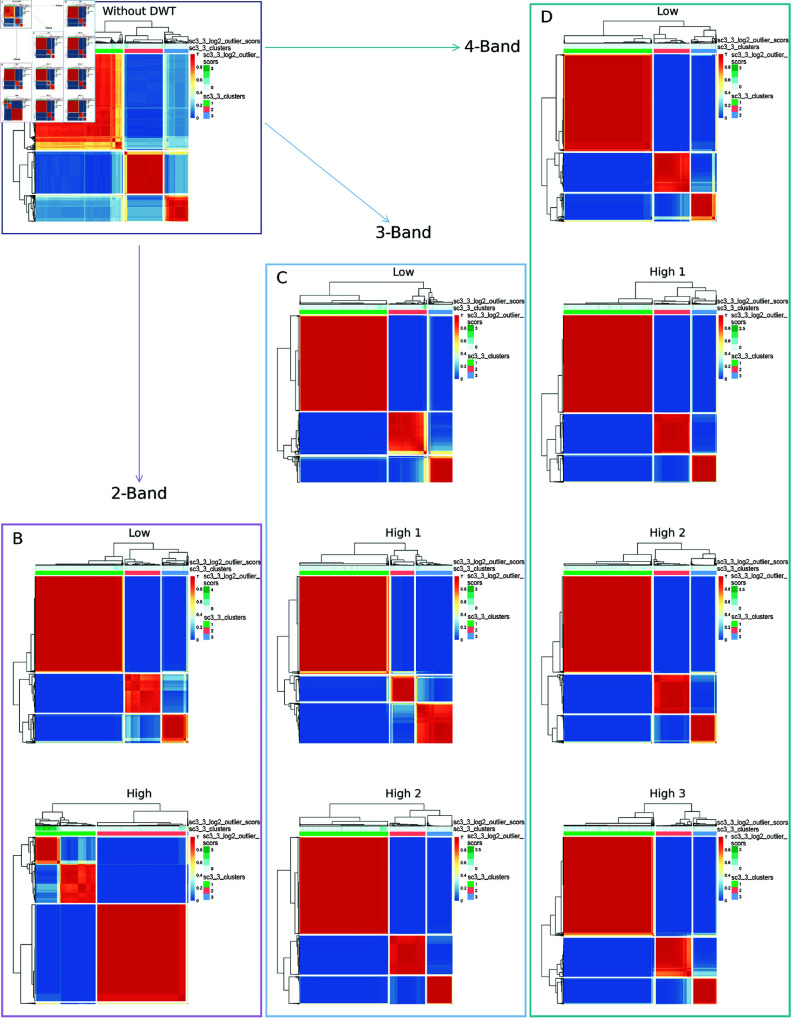
Consensus matrices among different cell phases of ILC differentiation dataset by Wavelet-SC3 method. **(A)** Consensus matrix using SC3 without DWT. ( **B**)-( **D**) are consensus matrices under Wavelet-SC3, with ( **B**) for 2-band DWT, ( **C**) for 3-band DWT, and ( **D**) for 4-band DWT.

In addition to the Wavelet-Seurat analysis, WMC is also performed on ILC differentiation cells dataset using the SC3 clustering method [25](Figs 3 and H in S1 Text). As shown in Fig 3, different wavelet components yield distinct hierarchical structures, with greater intra-cluster similarity observed in wavelet-transformed data compared to clustering without DWT.

To investigate cell-type-level resolution, we have employed Wavelet-SC3 to partition the dataset into 21 clusters per component, as determined by SC3 without DWT. As illustrated in Fig H in S1 Text, the approximation component under multi-band DWT closely mirrors the clustering pattern of the original dataset, whereas the detail components reveal additional, distinct cluster structures.

We further apply Wavelet-SC3 to the PBMC-3k dataset (Fig I in S1 Text). This dataset exhibits a complex hierarchical structure, with SC3 without DWT identifying 14 clusters. To evaluate whether Wavelet-SC3 improves clustering resolution compared to the original SC3 method, we perform clustering while fixing the number of clusters at 14, applying SC3 with and without wavelet transformation. Indeed, as shown in Fig I in S1 Text, it appears that the clustering pattern under 2-band DWT closely resembles the original SC3 clustering. However, with multi-band DWT, the approximation components enable more compact clusters captured with higher correlation, while the detail components are of additional, distinct cluster structures. Thus, Wavelet-SC3 has the capacity of enhancing better clustering resolution and uncovering finer-scale biological heterogeneity.

### Hierarchical analysis using Wavelet-HGC routine

**Fig 4:**
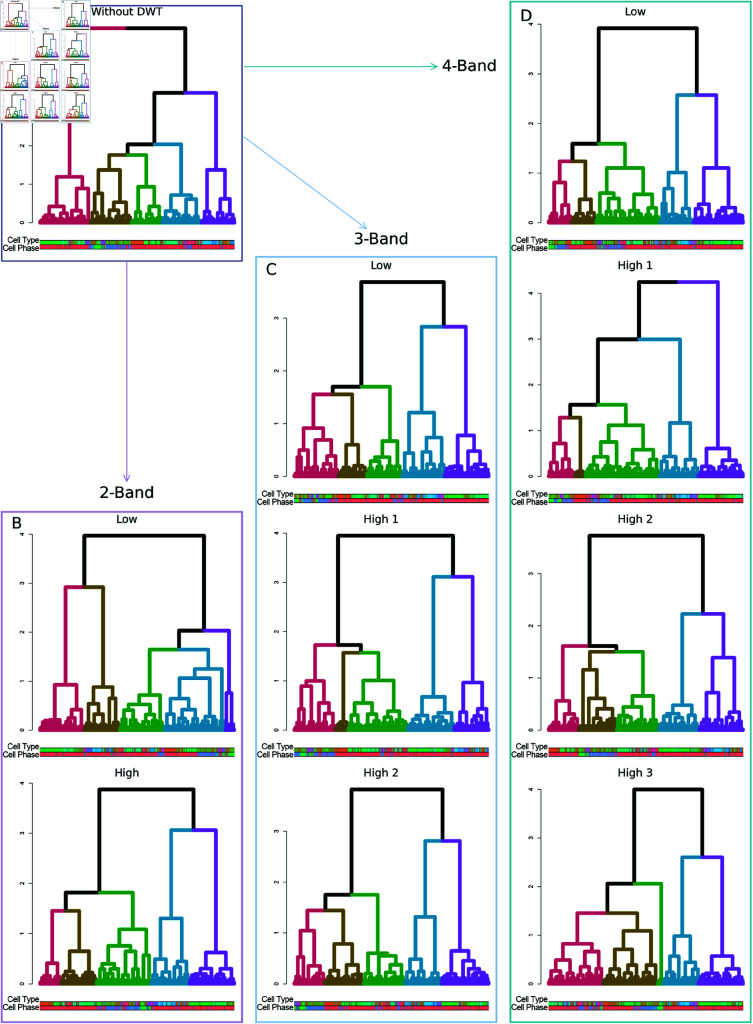
Multi-view hierarchical clusters on ILC dataset using Wavelet-HGC method. ( **A**) Dendrogram for ILC dataset without DWT. ( **B**)-( **D**) are dendrograms under wavelet analysis, with ( **B**) for 2-band DWT, ( **C**) for 3-band DWT, and ( **D**) for 4-band DWT.

To explore the hierarchical structure of clusters in scRNA-seq data, we have applied WMC in combination with the HGC method to the ILC differentiation dataset (Fig 4 )[24]. For cell cycle phases, we observe that 3-band DWT provides the most effective partitioning for G1-phase cells (marked in red), while detail components from 2-band and 4-band DWT successfully distinguish G2M-phase cells. In cell type clustering, the approximation component from 2-band wavelet-transformed data closely resembles the clustering structure obtained without DWT, whereas the detail components introduce alternative hierarchical structures, revealing additional biological insights.

Moreover, a similar pattern can be observed in the PBMC dataset using Wavelet-HGC (Fig J in S1 Text). Without DWT, HGC method yields nine cluster from the original data; under wavelet transformation, each component identifies eight or nine clusters. The low-resolution components provide an overview of major cell types, while the high-resolution components reveal finer-grained cellular structures, highlighting the multi-scale clustering capabilities of Wavelet-HGC.

### Distinct gene signatures of cell clusters from different wavelet-based multi-view windows

To quantify the improvement in multi-view clustering of cells from our algorithms, we assess the performance of clustering at two levels, overlapping’s of cells between clusters and intersections of gene markers.

We find 10 significant clusters using 2-band DWT for the breast cancer data, and one more cluster from detail component can be found when compared with data without DWT. However, the effects are marginal when utilizing multi-band DWT. Of note, increasing the number of intersections between clusters in different resolution components may result in some subsets containing a small number of cells with a relatively high p-value. We discover 22 significant clusters using 3-band DWT, where 10, 8 and 11 clusters are found in approximation, detail-1 and detail-2 component-based data, respectively. Similarly, a total of 28 significant clusters are revealed for 4-band DWT, with 10, 8, 9 and 9 clusters in approximation, detail-1, detail-2 and detail-3 component-based data, respectively. Consequently, one can use WMC to divide some clusters in original data into a few subgroups, resulting in the clusters with greater significance.

**Fig 5:**
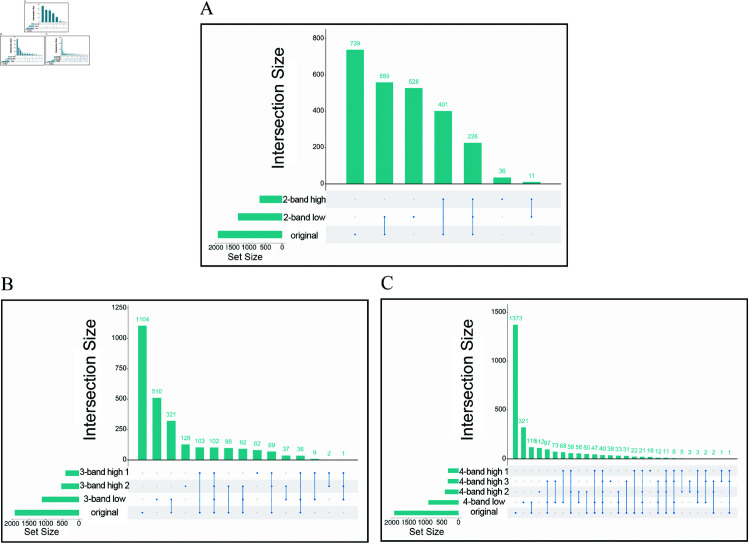
Assessing the performance of WMC on the breast cancer dataset. The x-axes of panels (A) through (C) represents the total number of genes in each component and the y-axes shows the number of genes in each intersection set. The cyan dots represent the number of genes belonging to the corresponding components, for gray dots vice versa. ( **A**) The intersection of genes between original data and its different wavelet transformed components using 2-band DWT, while ( **B**) for 3-band and ( **C**) for 4-band DWT, respectively.

Apparently, the cell types in different resolution components can overlap or be distinct. Indeed, comprehensive intersection analysis of gene signatures further support the power of multi-view of WMC (Fig 5).

Daubechies wavelet family, belonging 2-band wavelet families, produced one low-resolution component and one high-resolution component. Low-resolution parts are traditionally considered as an approximation of original data and share a number of common genes with original data. We find 528 more genes in the 2-band approximation component-based data than in original data (Fig 5A). Similarity, we discover 510 additional genes in 3-band approximation component-based data compared to original data (Fig 5B), and 321 more genes in 4-band approximation[0pc][-1pc]Figure 5 - The font size of the image is below 6 pt which affects the readability of the image. Hence, please supply a corrected version with font size above 6 pt. component (Fig 5C) than in original data.

DWT has the ability to decompose original data into orthogonal components, which is another important property. This means that different resolution parts are not overlapped theoretically. Using 2-band DWT, there are 237 genes that overlap in both resolution components, but only 11 (4.6%) of them are new, and the remaining 226 genes appeared in the original data (Fig 5A).

High-resolution components are particularly valuable for identifying cell types, as they capture fine-grained characteristics that may be overlooked in traditional analyses. Compared to the original dataset, the detail components extract through multi-band DWT reveal a greater number of cell-type-specific gene markers (Fig 5A–5C), underscoring the potential of wavelet transformation for enhancing marker gene detection.

Similar phenomena can be found in colorectal and PBMC dataset. In PBMC dataset, there are 133 genes that are identified at the intersection of two resolution components using 2-band DWT, whereas only 2 genes are absent from original data(Fig N in S1 Text). For the colorectal dataset, we only discover 80 at the intersection between two components using 2-band DWT, which is significantly less than the number of new genes only appearing in one single component. For example, we discover 301 and 201 genes in 2-band approximation and detail component-based data respectively (Fig O in S1 Text).

To further assess the consistency of clustering across wavelet-transformed components, we compute ARI and NMI as summarized in Table 1. The ARI values range from 0.6 to 0.8, while NMI values range from 0.57 to 0.73, indicating a moderate level of agreement. Notably, both ARI and NMI decrease as the number of wavelet bands increases from 2-band to 4-band DWT. It appears that the diversity of clustering outcomes introduced by multi-band wavelet transformation increases.

**Table 1:** Average ARI and NMI for clusters of M-band DWT components

The type of DWT	components	ARI	NMI
2-band	low vs high	0.80	0.73
3-band	low vs h1	0.72	0.65
3-band	low vs h2	0.76	0.70
3-band	h1 vs h2	0.73	0.67
4-band	low vs h1	0.66	0.59
4-band	low vs h2	0.61	0.57
4-band	low vs h3	0.64	0.58
4-band	h1 vs h2	0.69	0.63
4-band	h1 vs h3	0.73	0.65
4-band	h2 vs h3	0.65	0.61

**Fig 6:**
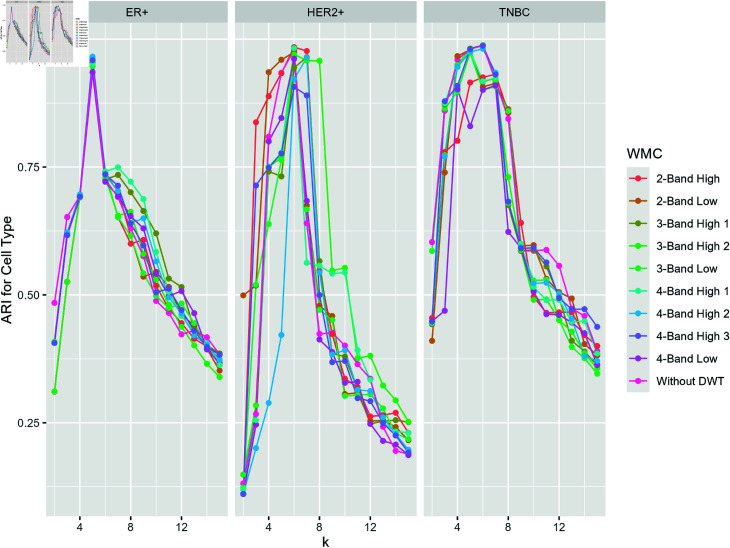
Average ARI results for breast cancer datasets on different subtypes of cancer cells.

In Fig 6, we present the compared average ARI across breast cancer datasets on performance of clustering methods with and without DWT. The detail components extracted via DWT consistently improve clustering accuracy across all breast cancer subtypes. The highest ARI values are observed for the 4-band detail component in ER+ (0.96) and TNBC (0.98) subtypes, while the 2-band detail component achieves the highest ARI for HER2+ (0.97). These results demonstrate the strong compatibility of WMC with breast cancer classification. Thus, WMC is robust in multi-view clustering.

## Discussion

In this study, we develop WMC, an M-band DWT-based approach for presenting scRNA-seq matrices via multi-view clustering of cells. WMC not only offers a unique capability that overcomes key limitations of existing single-cell analysis techniques, but also helps to identify missing cell types and rare cell types alongside activity. WMC can help discover rare cell types missed by conventional method with a fine resolution. Finally, WMC can potentially prioritize rare cell types for experimental validation by incorporating intersection analysis on multi-view of clusters.

Our study has certain limitations, as the current WMC framework is implemented with a maximum of 4-band DWT, balancing computational efficiency and multi-view clustering performance. It is plausible that using more than four bands could further enhance multi-scale clustering resolution. A key open question is determining the optimal choice of DWT type and band number for different biological datasets. We aim to systematically address this challenge through clustering optimization strategies in future work. For general WMC applications, the Daubechies (2-band) wavelet basis is recommended as the preferred option due to its computational efficiency and ease of implementation.

Moreover, even though multi-view clusters can detect unidentified cell types that may represent novel cell types in the biological field, these cell types are yet to functionally validate in a biological experiment. For future research, we envision that DWT-based framework integrated with other omics information is promising. Meanwhile, WMC has a great potential to analysis spatial single-cell gene expression data. Thus, our WMC paves the way to deploy wavelet tools in scRNA-seq data analysis.

## Materials and methods

### Data preprocessing

Any type of high-dimensional single-cell data can be transformed using WMC. However, before analysing the single-cell gene expression data, the raw scRNA-seq data often necessitates particular preprocessing and normalization to ensure that technical effect does not distort downstream analysis results. Given a raw data generated by sequencing machine, the quality control preformed based on following three criteria: 1) the number of counts per barcode (count depth), 2) the number of genes per barcode, and 3) the proportion of counts from mitochondrial genes per barcode. Since low-quality cells or empty droplets frequently have very few genes and a large number of detected genes may represent doublets, we begin by filtering cells with unique genes detected in excess of 3,000 or fewer than 200. Next, we filter out genes that are not expressed in fewer than 20 cells. In addition, cells with a mitochondrial count greater than 5 percent are removed, as cells with a relatively high mitochondrial count may be implicated in respiratory processes. We next perform a log normalization on the quality-controlled data matrix to reduce the variability of data before applying WMC.

### Discrete wavelet analysis

In our method to deal with the scRNA-seq data matrix, we use M-band DWT to decompose the count matrix (denoted by *S*) into M different resolution components, as shown in (3). In this paper, we select M=2,3, and 4, respectively, where the case of *M* = 2 corresponds to Daubechies wavelets family. The decomposition can be performed using the DWT matrix (see S1 Text for more details). The DWT projects the data into orthogonal subspaces with different resolutions, allowing us to extract the information partially hidden by the original data matrix. The wavelet-based filters satisfy a set of orthogonal condition. Thus, the DWT preserves the energy of the data since the *l*_2_-norm remains unchanged under orthonormal transform.

### Assessment using intersection analysis

We assess the performance of WMC via computing the intersection of $ℐ(S),ℐ(A),...,ℐ(D_{M-1})$, where ℐ(X) denotes that the data X is transformed by PCA and UMAP. Without wavelet transform, ℐ(S) gives a single-view clustering, while $ℐ(A),ℐ(D_{1}),...,ℐ(D_{M-1})$ provide a multi-view clustering of RNA-seq data. To examine the number of total clusters in multi-view windows, we calculate the distribution of significant genes among different resolutions components. Specifically, let *G*_0_ be the set of significant genes in the raw data, *G*_1_ be that in the approximation component, and $G_{2},...,G_{M}$ be that in the detail components, respectively. For each non-empty subset, Λ⊂{0,1,...,M}, we calculate $N_{\Lambda }=(\cap_{i\in \Lambda }G_{i})\cap (\cap_{j\notin \Lambda }{\bar{G}}_{j})$ i.e., the set of genes in the selected components but not in the other components. If |Λ|=1, $\left|N_{\Lambda }\right|$ denotes the number of exclusive genes in the corresponding component. Note that the components of different resolutions under the DWT are orthogonal to each other, if |Λ|≥2 and 0∉Λ, only a few genes will contain in such $N_{\Lambda }$ theoretically. The smaller number of $\left|N_{\Lambda }\right|$ for such intersection yields a better resolution of clustering.

### Adjusted rand index and normalized mutual information

The ARI is a widely used metric for quantifying the similarity between two clustering schemes. Let *n*_*ij*_ denote the number of cells in cluster *i* of scheme *A* and cluster *j* in scheme *B*. The ARI between clustering schemes *A* and *B* is computed as follows:

ARI(A,B)=∑i,j(nij2)−(∑i(ni+2)∑j(n+j2))/(N2)(∑i(ni+2)+∑j(n+j2))/2−(∑i(ni+2)∑j(n+j2))/(N2) (8)

where $n_{i+}=\sum_{j}n_{ij}$ represents the number of cells in cluster *i* of scheme *A*, $n_{+j}=\sum_{i}n_{ij}$ represents the number of cells in cluster *j* of scheme *B*, and *N* is the total number of cells.

In this study, ARI is utilized for two primary purposes: (1) evaluating the performance of WMC by comparing ARI values between WMC and the corresponding conventional framework without wavelet transformation; (2) assessing clustering consistency across different wavelet-transformed components, by computing ARI values for clustering results obtained from distinct resolution components.

ARI values range from -1 to 1, where a value closer to 1 indicates a high degree of similarity between clustering schemes, while a negative value suggests poor agreement and mismatching.

Another widely used metric for quantifying clustering similarity is the NMI, defined as:

$$NMI(A,B)=\frac{\sum_{i,j}\frac{n_{ij}}{N}\log\frac{n_{ij}N}{n_{i+}n_{+j}}}{\sqrt{\sum_{i}n_{i+}\log\frac{n_{i+}}{N}\sum_{j}n_{+j}\log\frac{n_{+j}}{N}}}.$$ (9)

The NMI score ranges from 0 to 1, where 1 indicates a perfect correspondence between the two clustering schemes. In this study, NMI is also employed to evaluate the consistency of multi-view clustering results across different wavelet components, providing insights into the robustness and diversity of the clustering outcomes.

## Supporting information

S1 Text Implementation details and supplementary results. This file provides detailed mathematical background on the DWT, implementation notes for the WMC framework, and additional results that support and extend the main findings presented in the manuscript
(PDF)
