# Supplementary material for: M-band wavelet-based multi-view clustering of cells
Source: PLoS Comput Biol. 2025 May 23;21(5):e1013060. doi: 10.1371/journal.pcbi.1013060 (PMC12143518; doi:10.1371/journal.pcbi.1013060)
Supplement: S1 Text — This file provides detailed mathematical background on the DWT, implementation notes for the WMC framework, and additional results that support and extend the main findings presented in the manuscript (PDF) [file pcbi.1013060.s001.pdf]

S1 Text: Supplementary Material for "M-Band Wavelet-Based Multi-view Clustering of Cells"

Tong Liu[a], Zihuan Liu[b], Wenke Sun[c], Adeethyia Shankar[d], Yongzhong Zhao[e], Xiaodi Wang[f]

a Department of Mathematical Sciences, Tsinghua University, Beijing, China

b Data and Statistical Science, AbbVie, Chicago, Illinois, United States

c School of Economics and Management, Dalian University of Technology, Dalian, China

d Brown University, Providence, Rhode Island, United States

e Frontage Labs, Exton, Pennsylvania, United States

f Department of Mathematics, Western Connecticut State University, Danbury, Connecticut, United States

A. Mathematical theory for DWT

An Orthogonal M-Band Discrete Wavelet Transform (DWT) is totally determined by M sets of filters in a filter bank with certain properties[S1,S2,S3]. In any such filter bank, there are one low pass filter α, and $M-1$ high pass filters $\beta^{(j)}$ for $j=1,\dots,M-1$ with N vanishing moments. These filters satisfy the following conditions:

$$\sum_{i=1}^{n} \alpha_i = \sqrt{M}, \tag{S1}$$

$$\sum_{i=1}^{n} i^k \beta_i^{(j)} = 0 \text{ for } k = 0,1,\dots,N-1, j = 1,\dots M-1, \tag{S2}$$

$$\|\alpha\| = \left\|\beta^{(j)}\right\| = 1 \text{ for } j = 1,\dots,M-1, \tag{S3}$$

$$\langle \alpha, \beta^{(j)} \rangle = 0 \quad \text{for } j = 1,\dots,M-1, \tag{S4}$$

$$\langle \beta^{(i)}, \beta^{(j)} \rangle = 0 \quad \text{for } i,j = 1,\dots,M-1 \text{ and } i \neq j. \tag{S5}$$

A M-Band DWT can be used to decompose a signal $S \in \mathbb{R}^{Mk}(k \in \mathbb{N}, k \geq M)$ into the sum of M different frequency components. To do so, we create a corresponding $Mk \times Mk$ DWT matrix W_1 by shifting and wrapping around the filters α and $\beta^{(j)}$ for $j=1,\dots,M-1$. It's not hard to verify that W_1 is an orthonormal matrix and therefore the column (row) vectors of W_1 form an orthonormal basis for $\mathbb{R}^{Mk}$. Then the first level DWT of S can be done by:

$$W_1 S = [a^1 \quad d_1^1 \quad d_2^1 \quad \dots \quad d_{M-1}^1]^T \triangleq \tilde{S}_1, \tag{S6}$$

where $a^1 = [a_1^1, a_2^1, \dots, a_k^1]^T$, and $d_i^1 = \left[d_{i,1}^1, d_{i,2,}^1 \dots, d_{i,k}^1\right]^T, i = 1,2,\dots,M-1.$

Intuitively, $W_1 S = \tilde{S}_1$ means that we transform S into the corresponding wavelet domain, or coordinates of S under the wavelet basis.

Generally, an M-Band L-regular DWT matrix has M filters with length ML, and the non-zero elements in each row shifts by M columns. To be concise, we show an example of 16×16 4-band 2-regular DWT matrix:

$$
\begin{bmatrix}
\alpha_1 & \alpha_2 & \alpha_3 & \alpha_4 & \alpha_5 & \alpha_6 & \alpha_7 & \alpha_8 & 0 & 0 & 0 & 0 & 0 & 0 & 0 & 0 \\
0 & 0 & 0 & 0 & \alpha_1 & \alpha_2 & \alpha_3 & \alpha_4 & \alpha_5 & \alpha_6 & \alpha_7 & \alpha_8 & 0 & 0 & 0 & 0 \\
0 & 0 & 0 & 0 & 0 & 0 & 0 & 0 & \alpha_1 & \alpha_2 & \alpha_3 & \alpha_4 & \alpha_5 & \alpha_6 & \alpha_7 & \alpha_8 \\
\alpha_5 & \alpha_6 & \alpha_7 & \alpha_8 & 0 & 0 & 0 & 0 & 0 & 0 & 0 & 0 & \alpha_1 & \alpha_2 & \alpha_3 & \alpha_4 \\
\beta_1 & \beta_2 & \beta_3 & \beta_4 & \beta_5 & \beta_6 & \beta_7 & \beta_8 & 0 & 0 & 0 & 0 & 0 & 0 & 0 & 0 \\
0 & 0 & 0 & 0 & \beta_1 & \beta_2 & \beta_3 & \beta_4 & \beta_5 & \beta_6 & \beta_7 & \beta_8 & 0 & 0 & 0 & 0 \\
0 & 0 & 0 & 0 & 0 & 0 & 0 & 0 & \beta_1 & \beta_2 & \beta_3 & \beta_4 & \beta_5 & \beta_6 & \beta_7 & \beta_8 \\
\beta_5 & \beta_6 & \beta_7 & \beta_8 & 0 & 0 & 0 & 0 & 0 & 0 & 0 & 0 & \beta_1 & \beta_2 & \beta_3 & \beta_4 \\
\gamma_1 & \gamma_2 & \gamma_3 & \gamma_4 & \gamma_5 & \gamma_6 & \gamma_7 & \gamma_8 & 0 & 0 & 0 & 0 & 0 & 0 & 0 & 0 \\
0 & 0 & 0 & 0 & \gamma_1 & \gamma_2 & \gamma_3 & \gamma_4 & \gamma_5 & \gamma_6 & \gamma_7 & \gamma_8 & 0 & 0 & 0 & 0 \\
0 & 0 & 0 & 0 & 0 & 0 & 0 & 0 & \gamma_1 & \gamma_2 & \gamma_3 & \gamma_4 & \gamma_5 & \gamma_6 & \gamma_7 & \gamma_8 \\
\gamma_5 & \gamma_6 & \gamma_7 & \gamma_8 & 0 & 0 & 0 & 0 & 0 & 0 & 0 & 0 & \gamma_1 & \gamma_2 & \gamma_3 & \gamma_4 \\
\delta_1 & \delta_2 & \delta_3 & \delta_4 & \delta_5 & \delta_6 & \delta_7 & \delta_8 & 0 & 0 & 0 & 0 & 0 & 0 & 0 & 0 \\
0 & 0 & 0 & 0 & \delta_1 & \delta_2 & \delta_3 & \delta_4 & \delta_5 & \delta_6 & \delta_7 & \delta_8 & 0 & 0 & 0 & 0 \\
0 & 0 & 0 & 0 & 0 & 0 & 0 & 0 & \delta_1 & \delta_2 & \delta_3 & \delta_4 & \delta_5 & \delta_6 & \delta_7 & \delta_8 \\
\delta_5 & \delta_6 & \delta_7 & \delta_8 & 0 & 0 & 0 & 0 & 0 & 0 & 0 & 0 & \delta_1 & \delta_2 & \delta_3 & \delta_4
\end{bmatrix},
$$

where

α= [-0.06737176, 0.09419511, 0.40580489, 0.56737176, 0.56737176, 0.40580489, 0.09419511, -0.06737176],

β= [-0.09419511,0.06737176,0.56737176,0.40580489, -0.40580489, -0.56737176, -0.06737176, 0.0941951],

γ= [-0.09419511, -0.06737176, 0.56737176, -0.4058048, -0.4058048, -0.5673717, -0.06737176, -0.0941951],

δ= [-0.06737176, -0.09419511,0.40580489, -0.56737176,0.56737176, -0.40580489, 0.09419511, 0.06737176]

are corresponding filters.

Let $C_1, C_2, \dots, C_{Mk}$ be the column vectors of W_1^T. Then, ${C_1}^T, {C_2}^T, \dots, {C_{Mk}}^T$ are row vectors of W_1. Since W_1 is a orthogonal matrix, $\{C_1, C_2, \dots, C_{Mk}\}$ forms an orthonormal basis of $\mathbb{R}^{Mk}$. Therefore, the components of $\tilde{S}_1$ are coordinates of *S* under this wavelet basis, and hence $\|W_1 S\| = \|\tilde{S}_1\|$. The components of $\tilde{S}_1$ are also called the wavelet coefficients of *S*. Moreover, the M-Band DWT of *S* decomposes *S* into *M* different frequency components with a^1 being the lowest frequency component (or trend) and $\mathrm{d}_1^1, \dots, \mathrm{d}_{\mathrm{M}-1}^1$ being the higher frequency components (or fluctuations) of *S*. If necessary, and *k* is divisible by *M*, we can apply DWT to a^1 using a $k \times k$ DWT matrix W_2 such that:

$$W_2 a^1 = [a^2 \quad \mathrm{d}_1^2 \quad \dots \quad \mathrm{d}_{M-1}^2]^T \triangleq \tilde{S}_2, \tag{S7}$$

where $a^2 = \left[a_1^2, a_{2,}^2 a_3^2, \dots, a_{\frac{k}{M}}^2\right]^{\mathrm{T}}$, $\mathrm{d}_{\mathrm{i}}^2 = \left[d_{i,1}^2, d_{i,2}^2, d_{i,3}^2, \dots, d_{i,\frac{k}{M}}^2\right]^T, i = 1, \dots M-1.$

The M-Band DWT of a^1 decomposes a^1 into M different frequency components with a^2 being lowest frequency and d_i^2 $(i = 1,\ldots, M-1)$ being higher frequency components of a^1.

Let W=$\begin{bmatrix} W_2 & \mathbf{0} \\ \mathbf{0} & I \end{bmatrix}$$W_1$, where the lower corner $\mathbf{0}$ is an $(M-1)k \times k$ zero matrix, upper corner $\mathbf{0}$ is a $k \times (M-1)k$ zero matrix, and I is an $(M-1)k \times (M-1)k$ identity matrix. Then

$$WS = [a^2 \quad \mathrm{d}_1^2 \quad \ldots \quad \mathrm{d}_{M-1}^2 \quad \mathrm{d}_1^1 \quad \ldots \quad \mathrm{d}_{M-1}^1]^T \triangleq \tilde{S}_2, \tag{S8}$$

and $\tilde{S}_2$is the second level wavelet coordinates of S. Since $\{C_1, C_2, \ldots, C_{Mk}\}$ is an orthonormal basis of $\mathbb{R}^{Mk}$,

$$S = s_1 C_1 + s_2 C_2 + \cdots + s_n C_n, \tag{S9}$$

where $n = Mk$ and $s_i = {C_i}^T S = \langle C_i, S\rangle$, the inner product of C_i and S for $i = 1,2,\ldots,n$. Therefore:

$$s_i = \begin{cases} a_i^1 \text{ for } i = 1,2,\ldots,k \\ d_{1,i}^1 \text{ for } i = (k+1), k+2, \ldots, 2k \\ \vdots \\ d_{(M-1),i}^1 \text{ for } i = (M-1)k+1, \ldots, Mk \end{cases}. \tag{S10}$$

Let:

$$\begin{cases} A^1 = a_1^1 C_1 + \cdots + a_k^1 C_k, \\ D_i^1 = d_{i,1}^1 C_{ik+1} + \cdots + d_{i,(i+1)k}^1 C_{(i+1)k} \end{cases} \tag{S11}$$

for $i = 1, \ldots, M-1$. Then A^1 is corresponding to a^1 and D_i^1 is corresponding to d_i^1 for $\mathrm{i} = 1, \ldots, M-1$.

If we let

$$\begin{gathered} \mathcal{V}_1 = span\{C_1, \ldots, C_k\}, \\ \mathcal{W}_1^{(i)} = span\{C_{ik+1}, \ldots, C_{(i+1)k}\}, \text{ for } i = 1, \ldots, M-1, \end{gathered} \tag{S12}$$

then $\mathcal{V}_1$, $\mathcal{W}_1^{(1)}, \ldots,$ and $\mathcal{W}_1^{(M-1)}$ are orthogonal to each other and therefore $\mathbb{R}^{Mk}$ can be represented as the following direct sum[S4]

$$\mathbb{R}^{Mk} = \mathcal{V}_1 \oplus \mathcal{W}_1^{(1)} \oplus \ldots \oplus \mathcal{W}_1^{(M-1)}. \tag{S13}$$

So, for any $S \in \mathbb{R}^{Mk}$, S can be written uniquely as[S4]

$$S = A^{(1)} + D_1^{(1)} + \cdots + D_{M-1}^{(1)}, \tag{S14}$$

where $A^{(1)} = Proj_{\mathcal{V}_1} S$ is the orthogonal projection of S onto $\mathcal{V}_1$, $D_i^{(1)} = Proj_{\mathcal{W}_i^1} S$ is orthogonal projection of S onto $\mathcal{W}_i^{(1)}$, for $\mathrm{i} = 1, \ldots, M-1$.

If we apply the second level DWT to S, then $\mathcal{V}_2$, $\mathcal{W}_2^{(1)}, \ldots, \mathcal{W}_2^{(M-1)}$, $\mathcal{W}_{1,}^{(1)} \ldots,$ and $\mathcal{W}_1^{(M-1)}$ are orthogonal to each other and therefore

$$\mathbb{R}^{Mk} = \mathcal{V}_2 \oplus \mathcal{W}_2^{(1)} \oplus \ldots \oplus \mathcal{W}_2^{(M-1)} \oplus \mathcal{W}_1^{(1)} \oplus \ldots \oplus \mathcal{W}_1^{(M-1)}, \tag{S15}$$

$$\mathcal{V}_1 = \mathcal{V}_2 \oplus \mathcal{W}_2^{(1)} \oplus \ldots \oplus \mathcal{W}_2^{(M-1)}, \tag{S16}$$

$$\mathrm{S} = A^{(2)} + D_1^{(2)} + \cdots + D_{M-1}^{(2)} + D_1^{(1)} + \cdots + D_{M-1}^{(1)}, \tag{S17}$$

$$A^{(1)} = A^{(2)} + D_1^{(2)} + \cdots + D_{M-1}^{(2)}. \tag{S18}$$

B. Supplementary Results

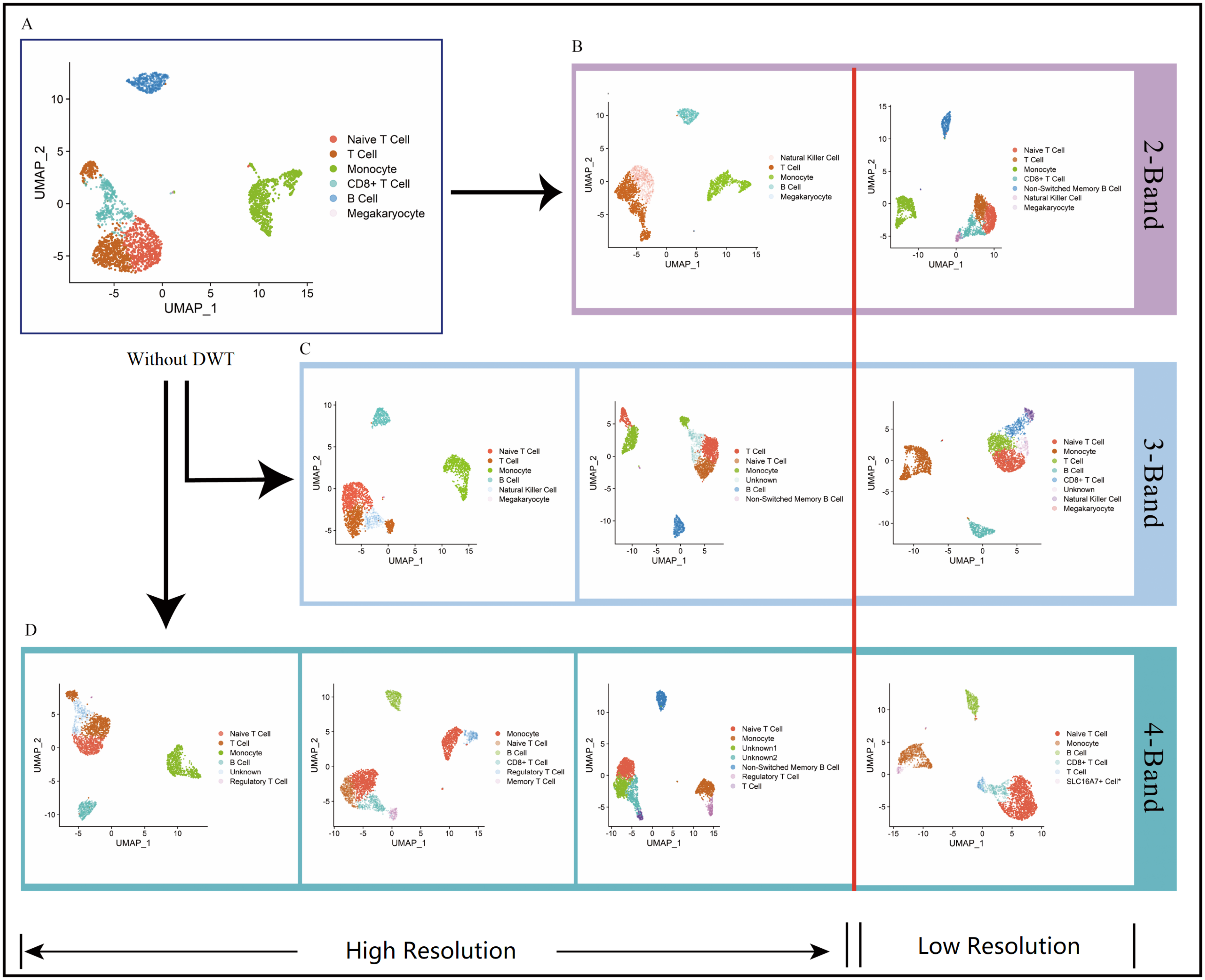

Fig A: Multi-view of clusters of PBMC dataset. (A) UMAP visualization of cell types based on data matrix without DWT. (B)-(D) are clusters under wavelet analysis, with (B) for 2-band DWT, (C) for 3-band DWT, and (D) for 4-band DWT.

*also might be mitotic arrest phase fetal germ cell

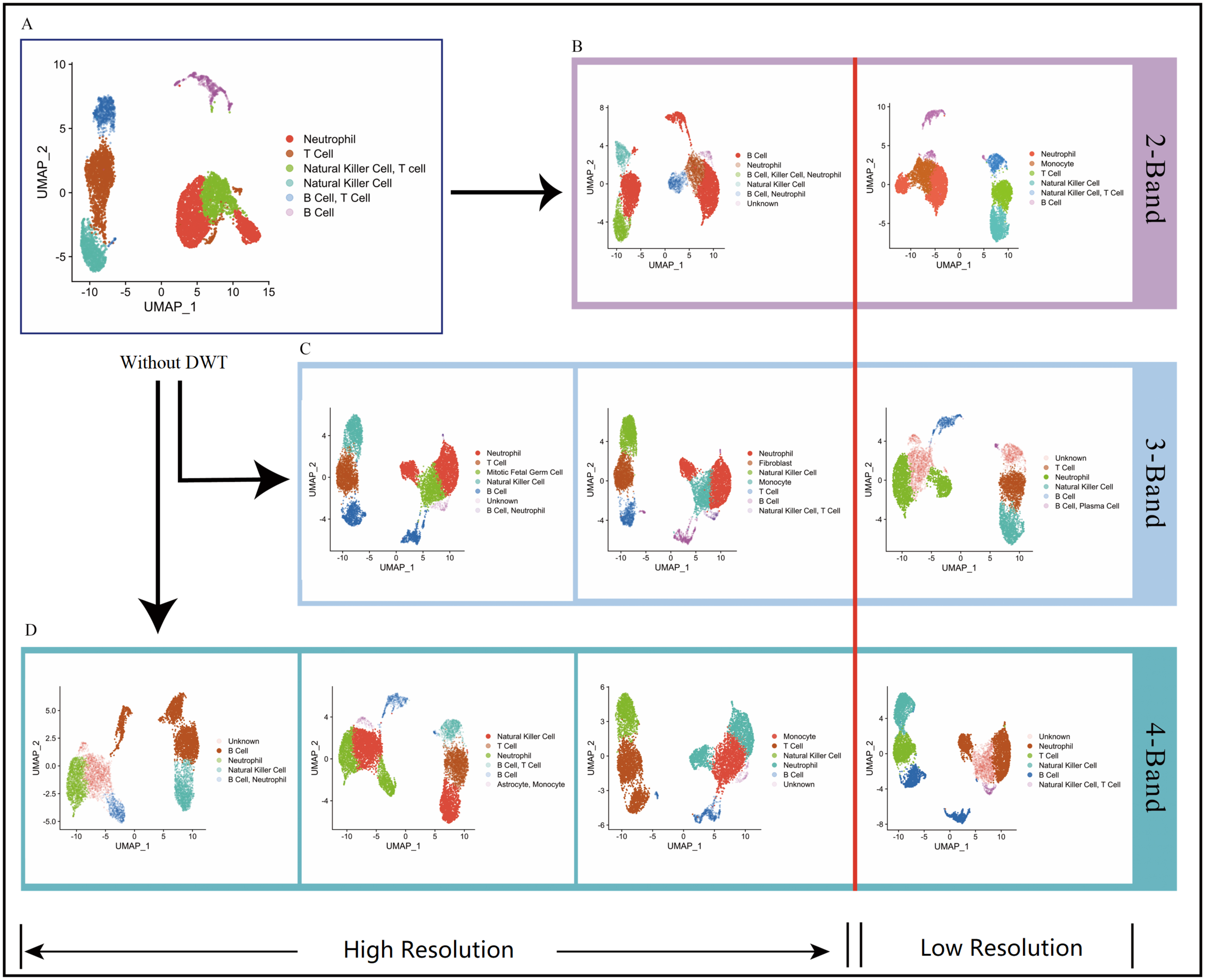

Fig B: Multi-view of clusters of colorectal dataset. (A) UMAP visualization of cell types based on data matrix without DWT. (B)-(D) are clusters under wavelet analysis, with (B) for 2-band DWT, (C) for 3-band DWT, and (D) for 4-band DWT.

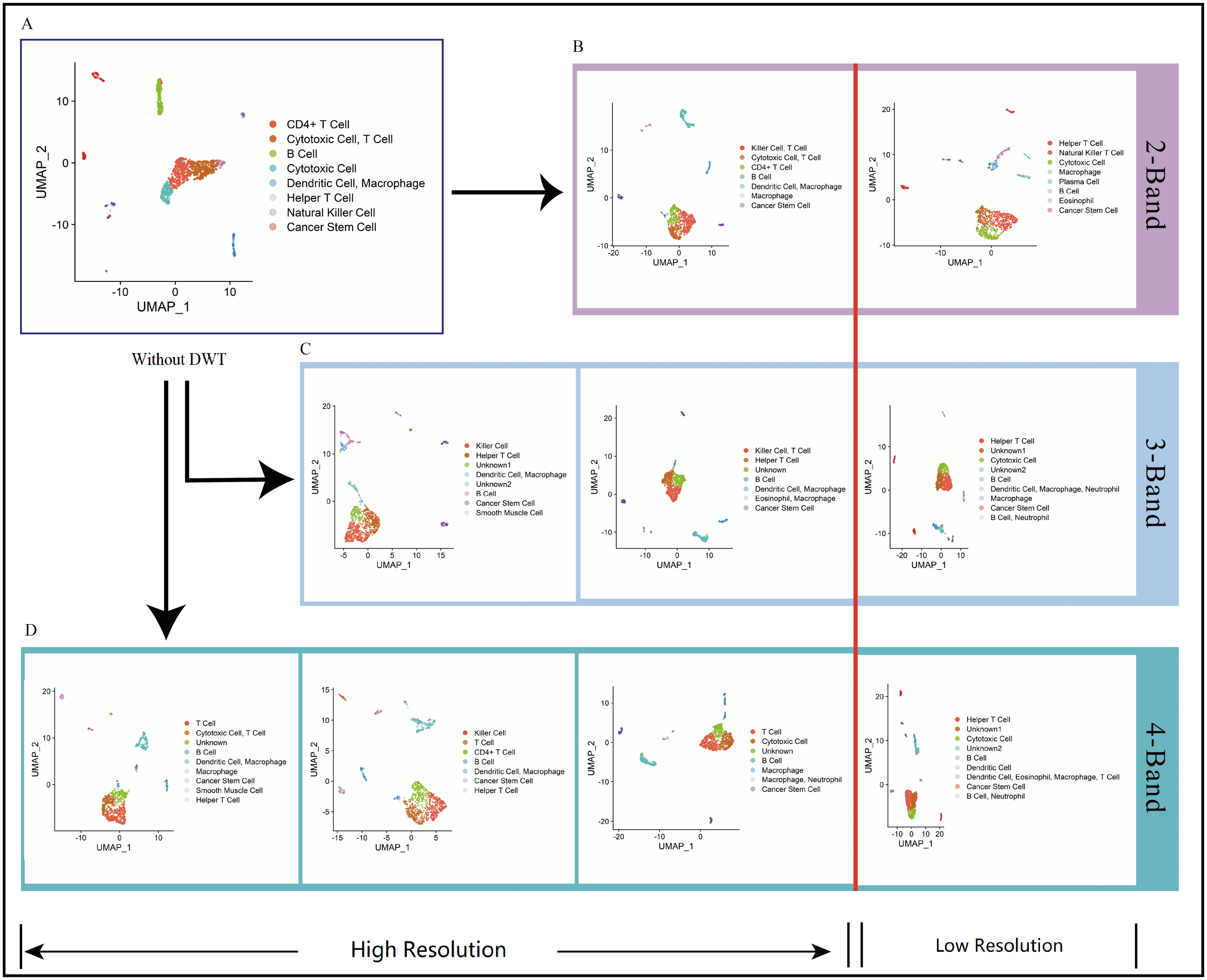

Fig C: Multi-view of clusters of dataset CID3921. (A) UMAP visualization of cell types based on data matrix without DWT. (B)-(D) are clusters under wavelet analysis, with (B) for 2-band DWT, (C) for 3-band DWT, and (D) for 4-band DWT.

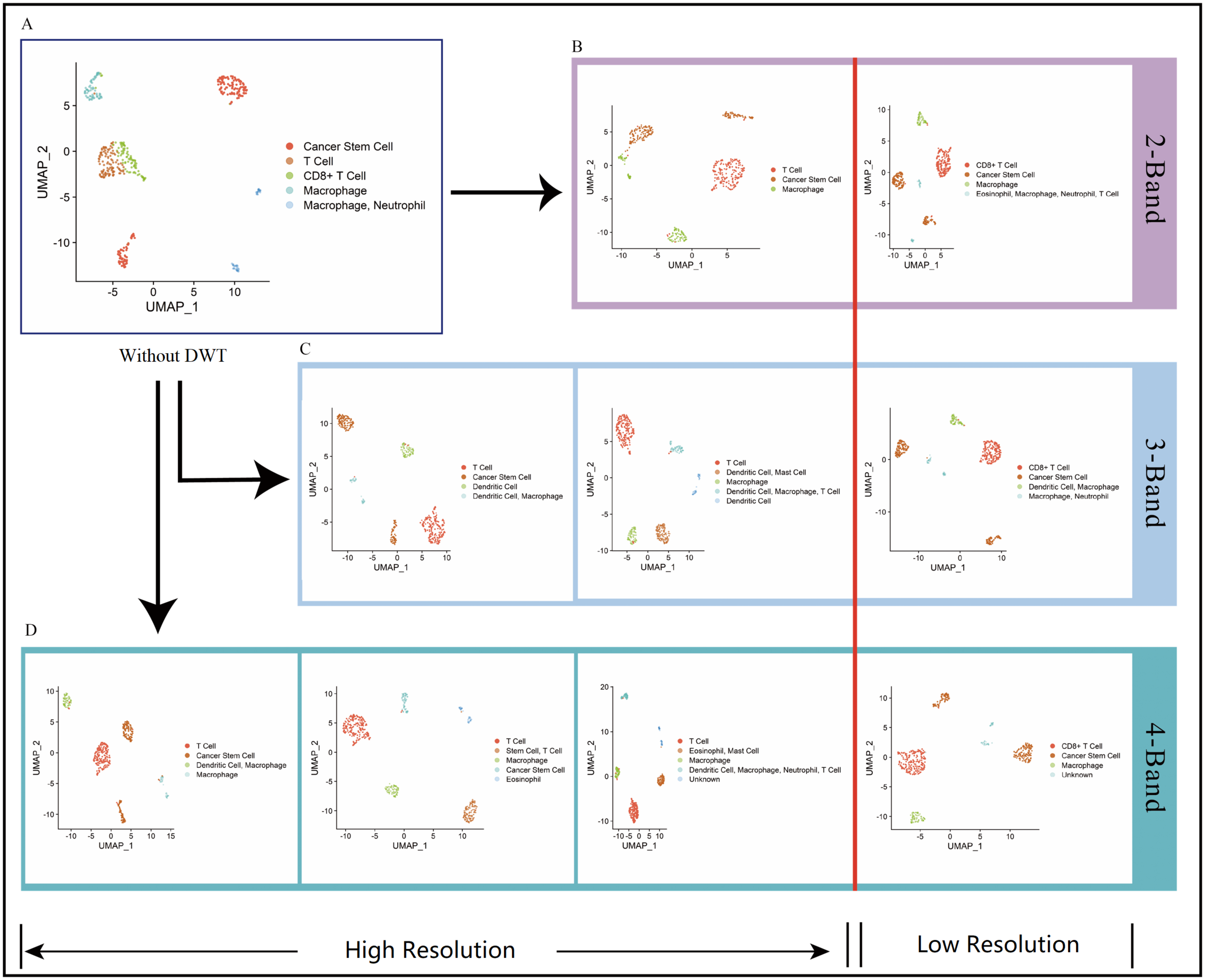

Fig D: Multi-view of clusters of dataset CID4463. (A) UMAP visualization of cell types based on data matrix without DWT. (B)-(D) are clusters under wavelet analysis, with (B) for 2-band DWT, (C) for 3-band DWT, and (D) for 4-band DWT.

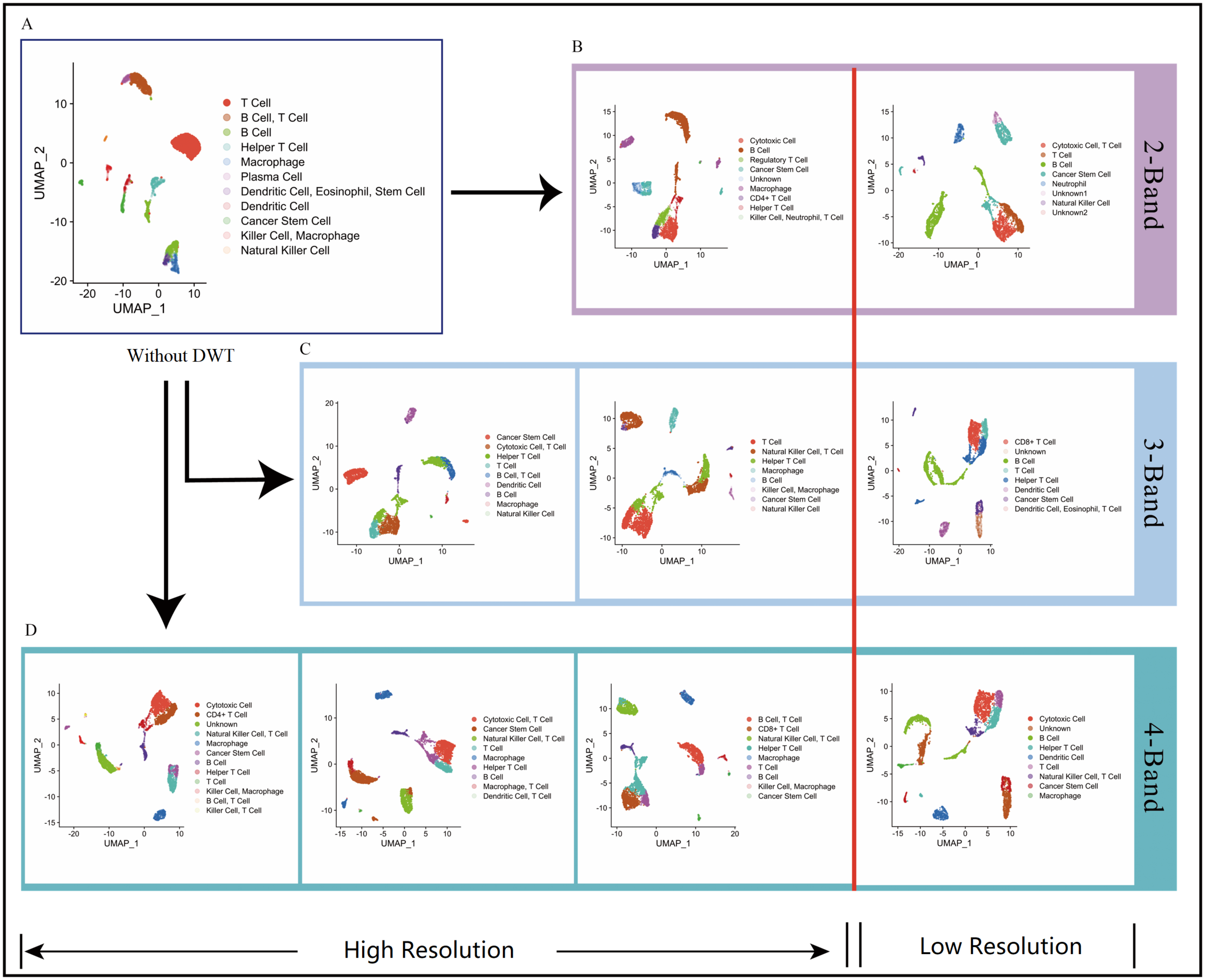

Fig E: Multi-view of clusters of dataset CID4495. (A) UMAP visualization of cell types based on data matrix without DWT. (B)-(D) are clusters under wavelet analysis, with (B) for 2-band DWT, (C) for 3-band DWT, and (D) for 4-band DWT.

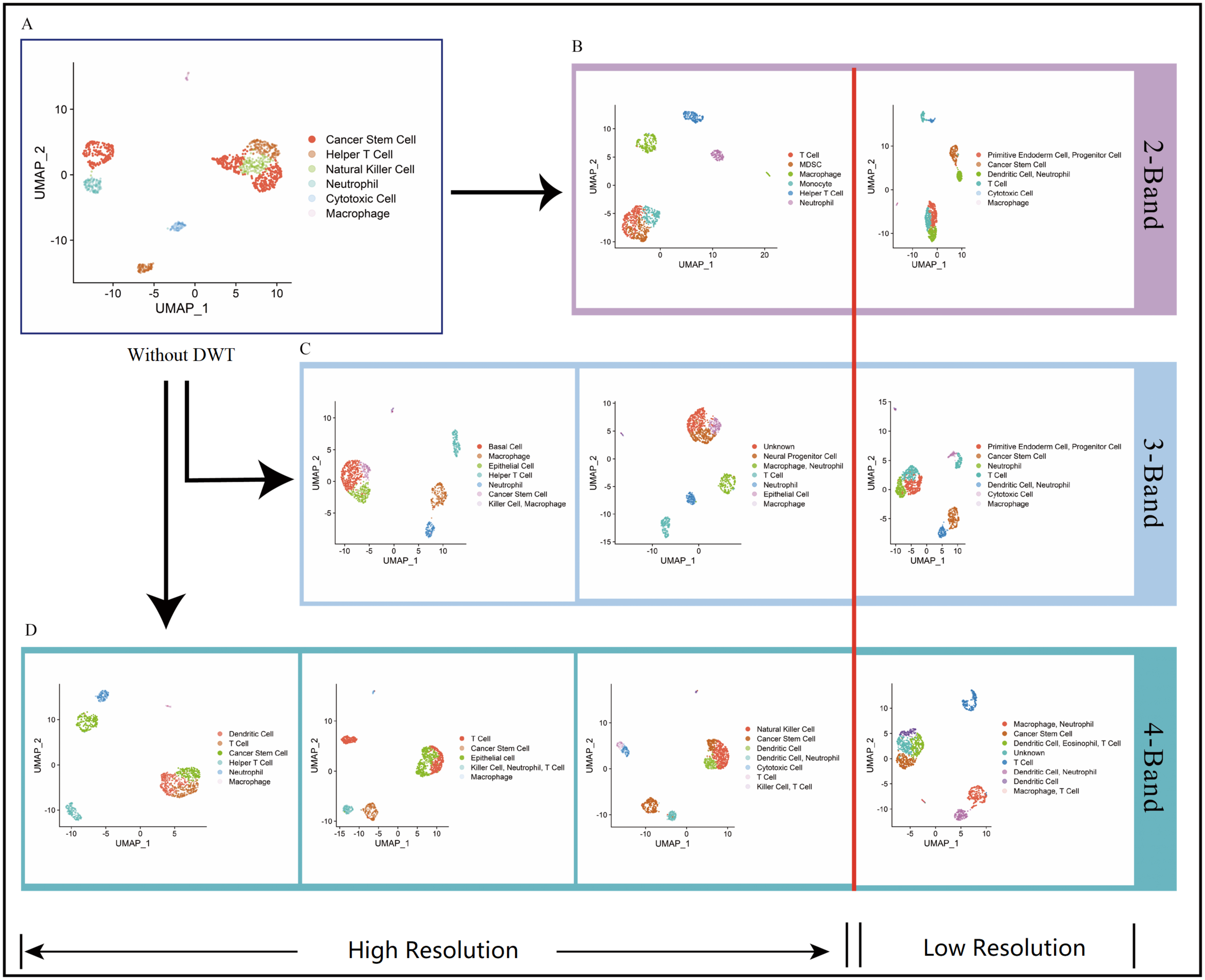

Fig F: Multi-view of clusters of dataset CID4523. (A) UMAP visualization of cell types based on data matrix without DWT. (B)-(D) are clusters under wavelet analysis, with (B) for 2-band DWT, (C) for 3-band DWT, and (D) for 4-band DWT.

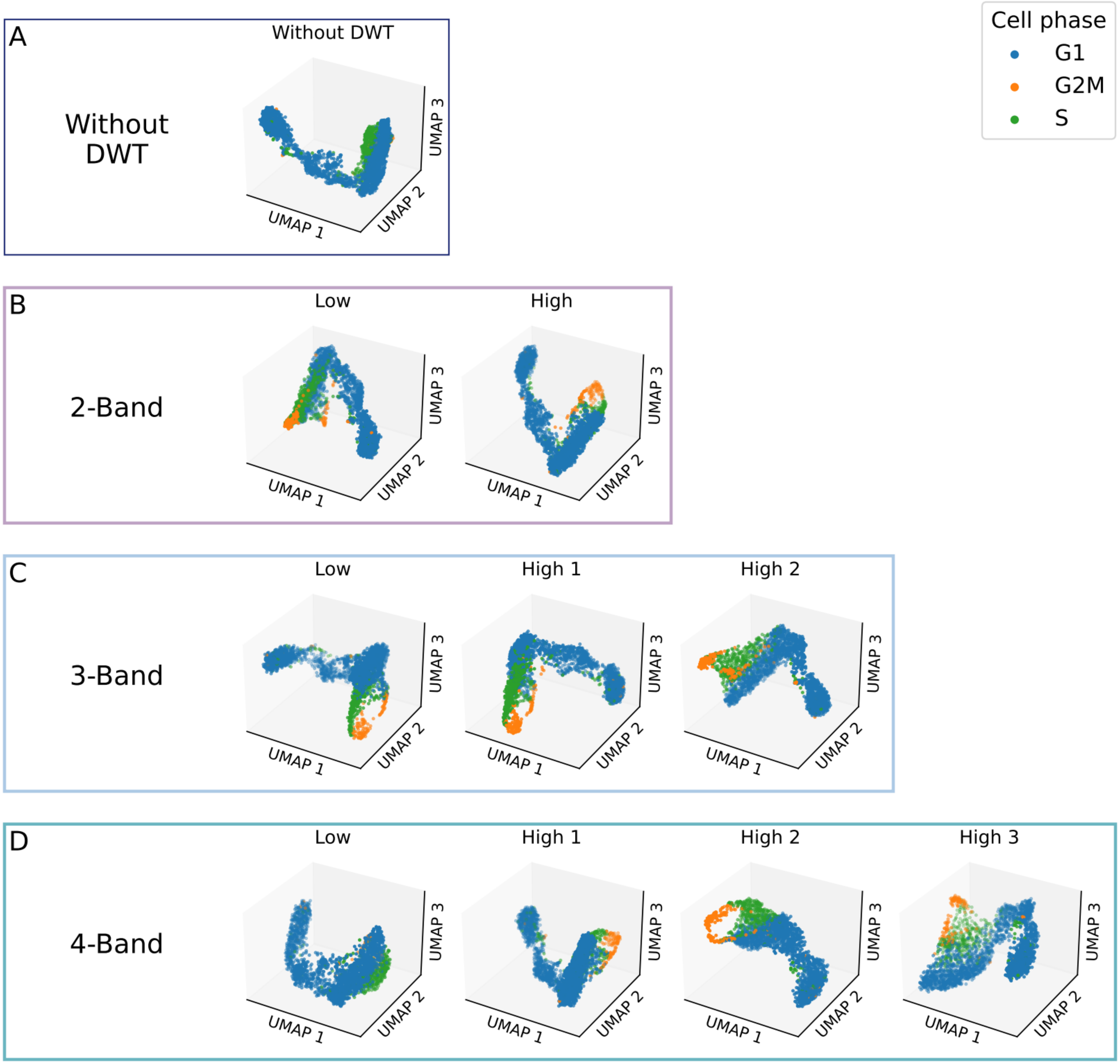

Fig G: Multi-view of cell phases for ILC dataset. (A) UMAP visualization of cell phases based on matrix without DWT. (B)-(D) are clusters under wavelet analysis, with (B) for 2-band DWT, (C) for 3-band DWT, and (D) for 4-band DWT.

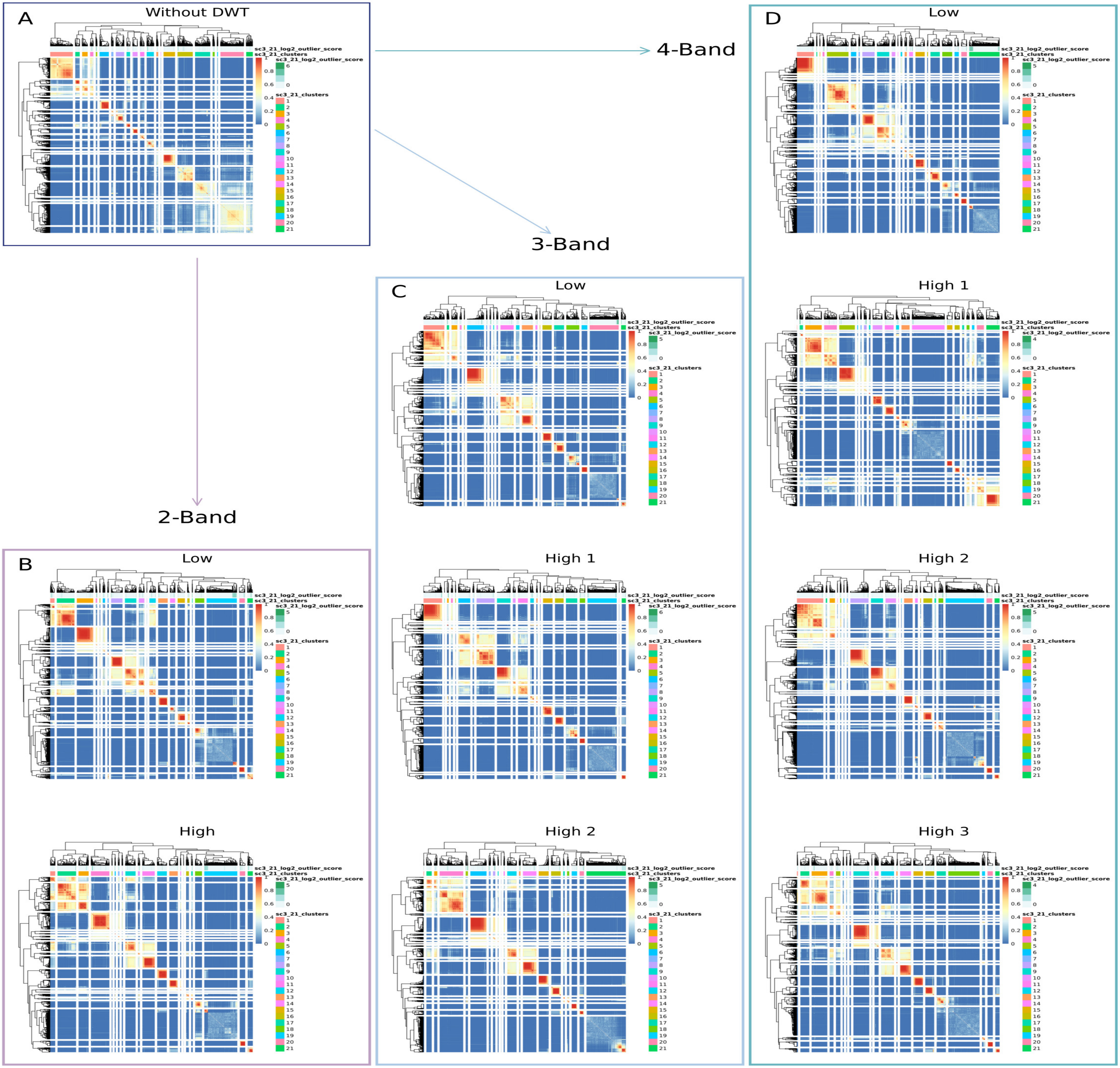

Fig H: Consensus matrix among different cell types of ILC dataset by Wavelet-SC3 methods. (A) consensus matrix using SC3 without DWT. (B)-(D) are consensus matrices under wavelet analysis, with (B) for 2-band DWT, (C) for 3-band DWT, and (D) for 4-band DWT.

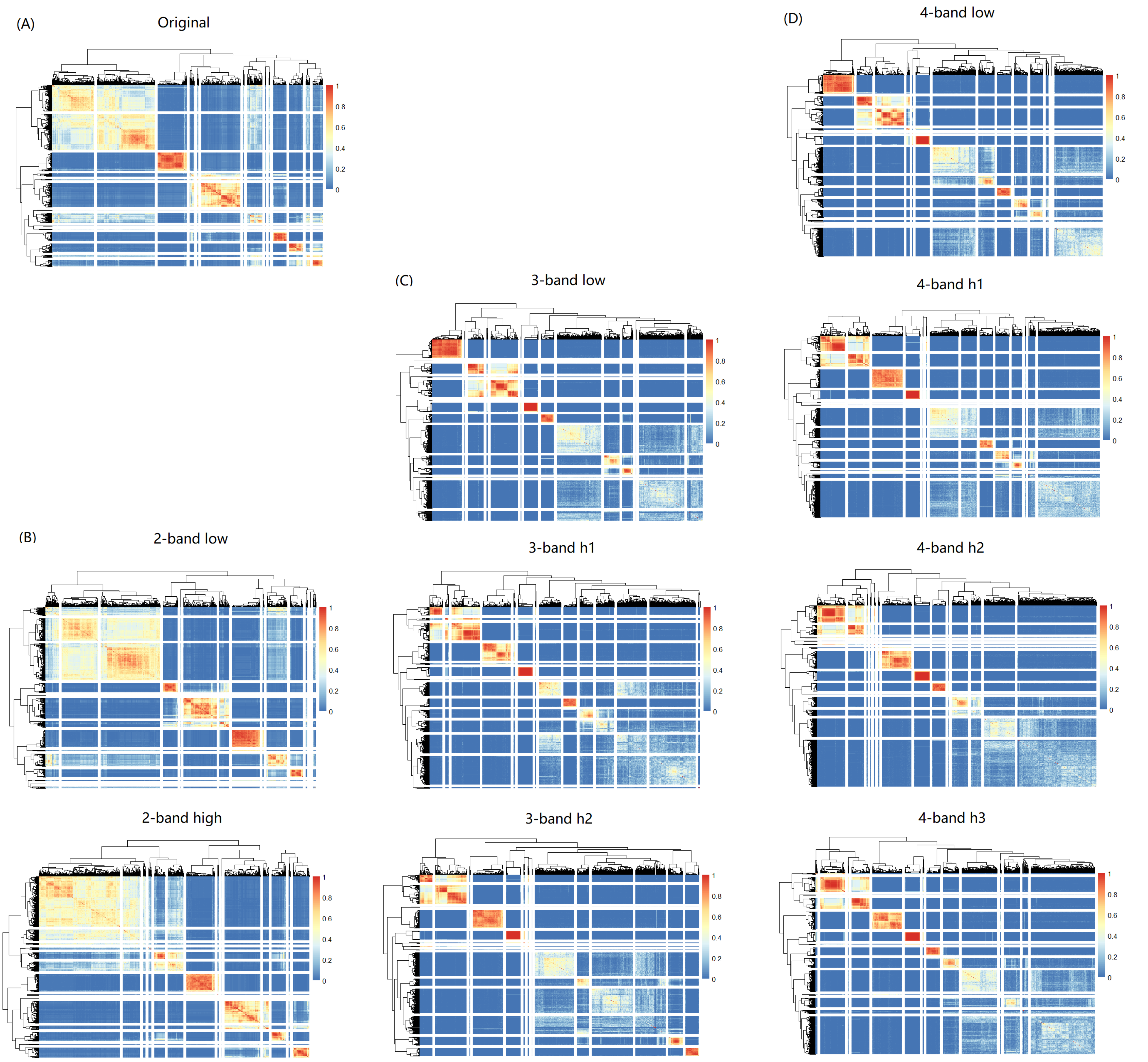

Fig I: Consensus matrix of PBMC dataset by Wavelet-SC3 methods. (A) consensus matrix using SC3 without DWT. (B)-(D) are consensus matrices under wavelet analysis, with (B) for 2-band DWT, (C) for 3-band DWT, and (D) for 4-band DWT.

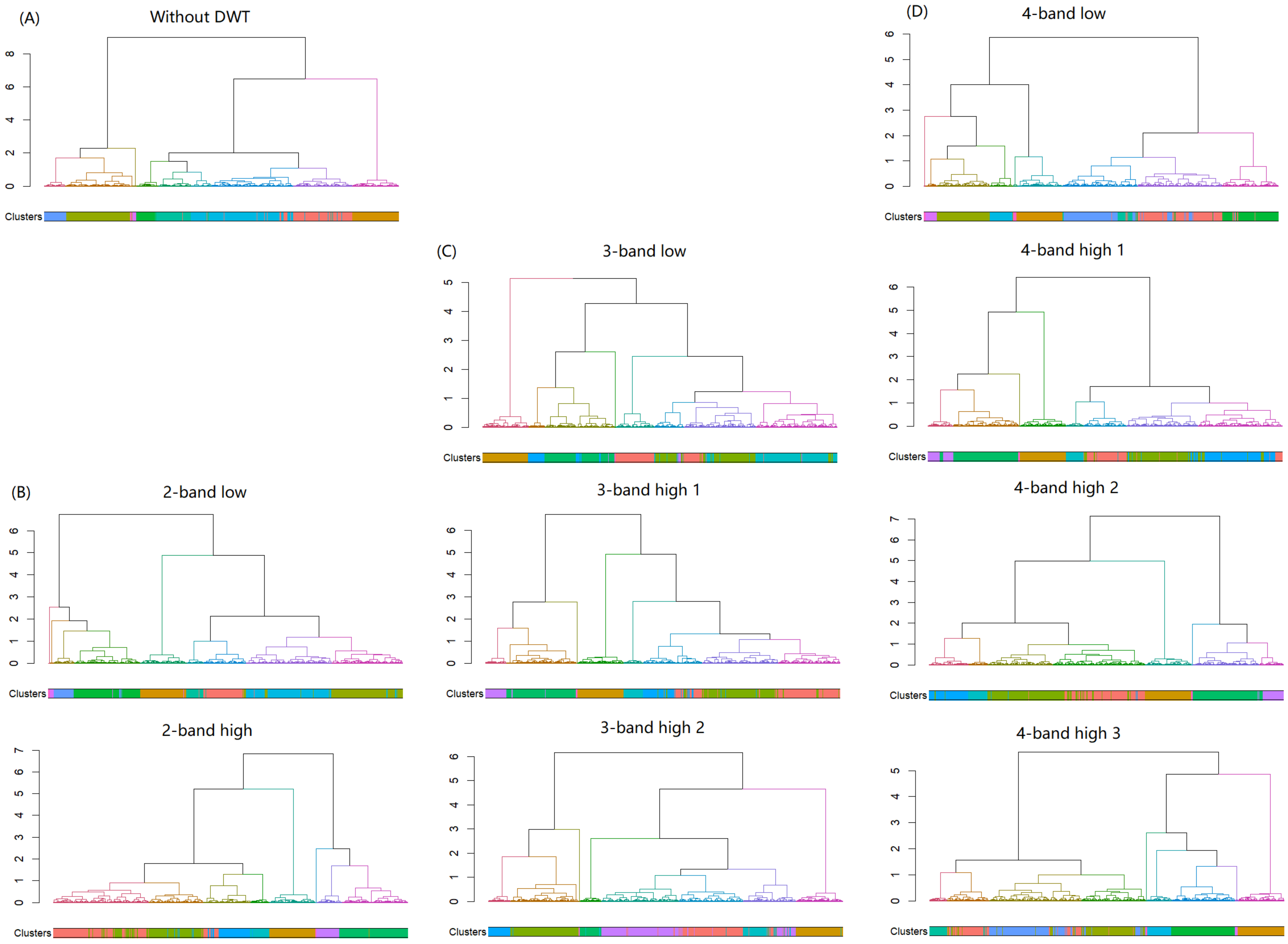

Fig J: Multi-view hierarchical clusters on PBMC dataset using Wavelet-HGC method. (A) Dendrogram for PBMC dataset without DWT. (B)-(D) are dendrograms under wavelet analysis, with (B) for 2-band DWT, (C) for 3-band DWT, and (D) for 4-band DWT.

In each of the following Fig K-O, The x-axis of panels represents the total number of genes in each component and the y-axis of panels shows the number of genes in each intersection set. The cyan dots represent the number of genes belonging to the corresponding components, for gray dots vice versa. Each sub-figure (A)-(C) analyses the intersection of genes in canonical data and its different frequency components, using 2-band, 3-band and 4-band DWT, respectively.

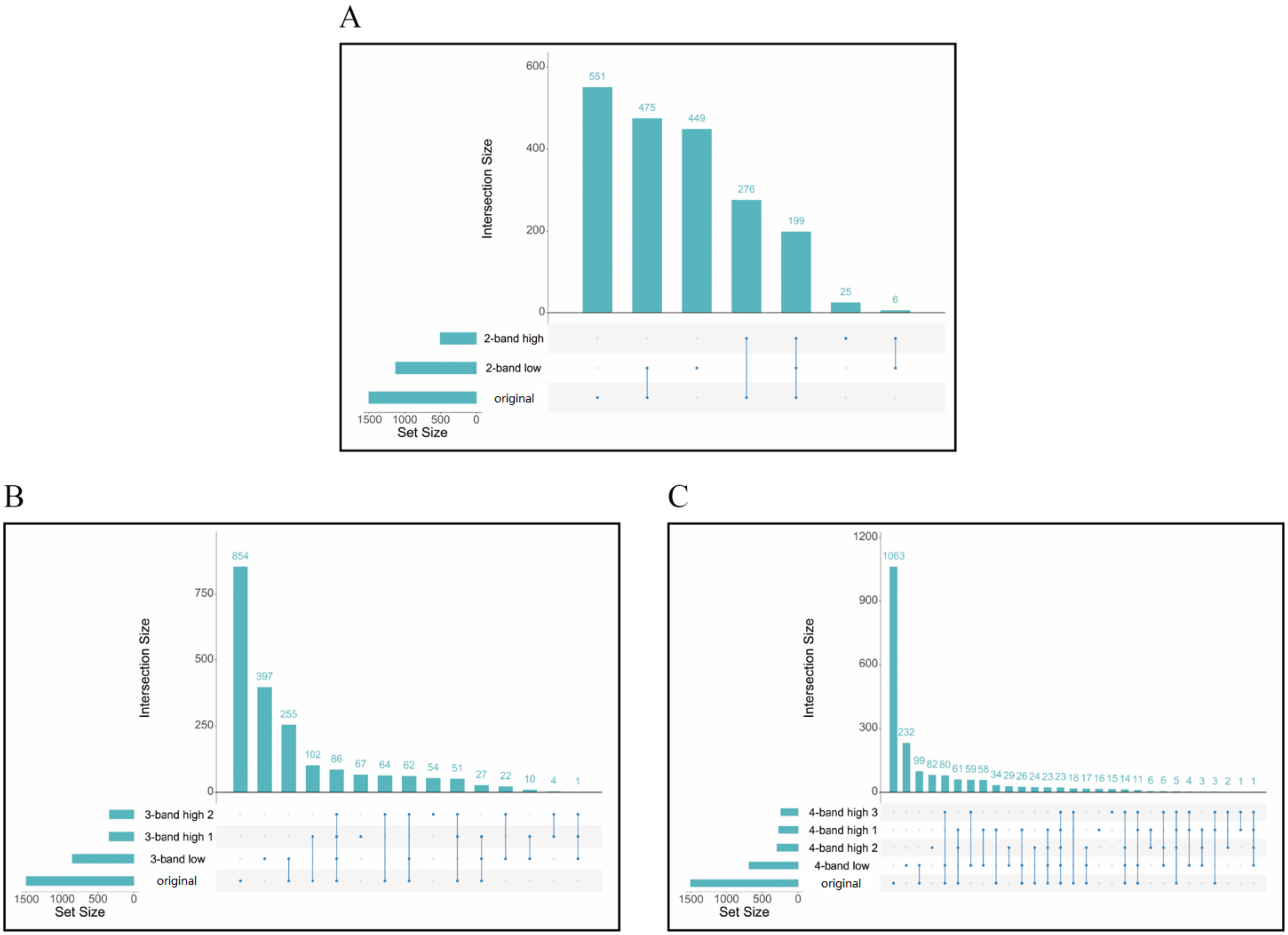

Fig K: The intersection of genes and cell-type related markers for CID4463. (A) The intersection of genes between canonical data and its different frequency components using 2-band DWT (Daub4). (B) Similar to (A), but using 3-band DWT. (C) Similar to (A), but using 4-band DWT.

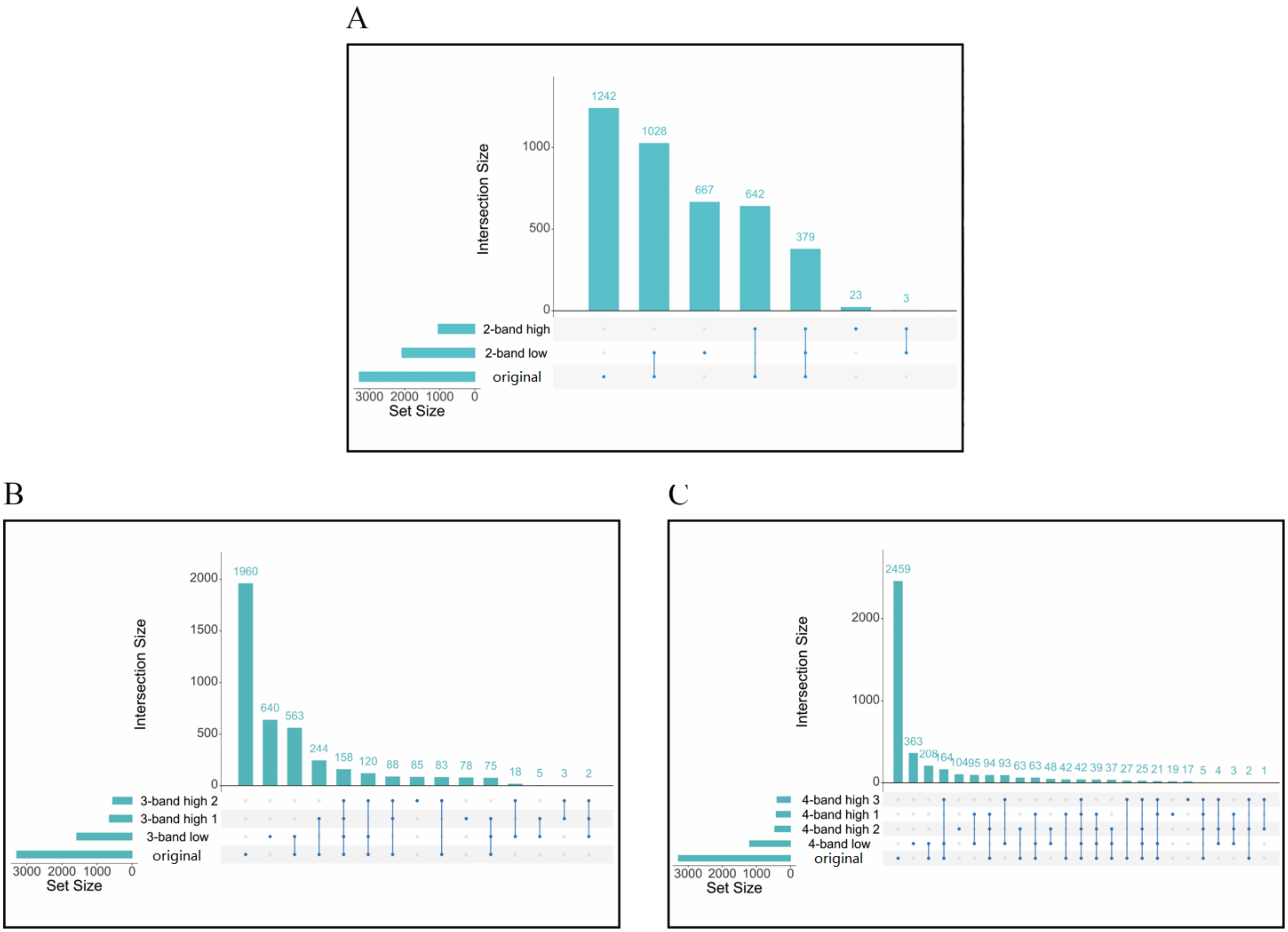

Fig L: The intersection of genes and cell-type related markers for CID4495. (A) The intersection of genes between canonical data and its different frequency components using 2-band DWT (Daub4). (B) Similar to (A), but using 3-band DWT. (C) Similar to (A), but using 4-band DWT.

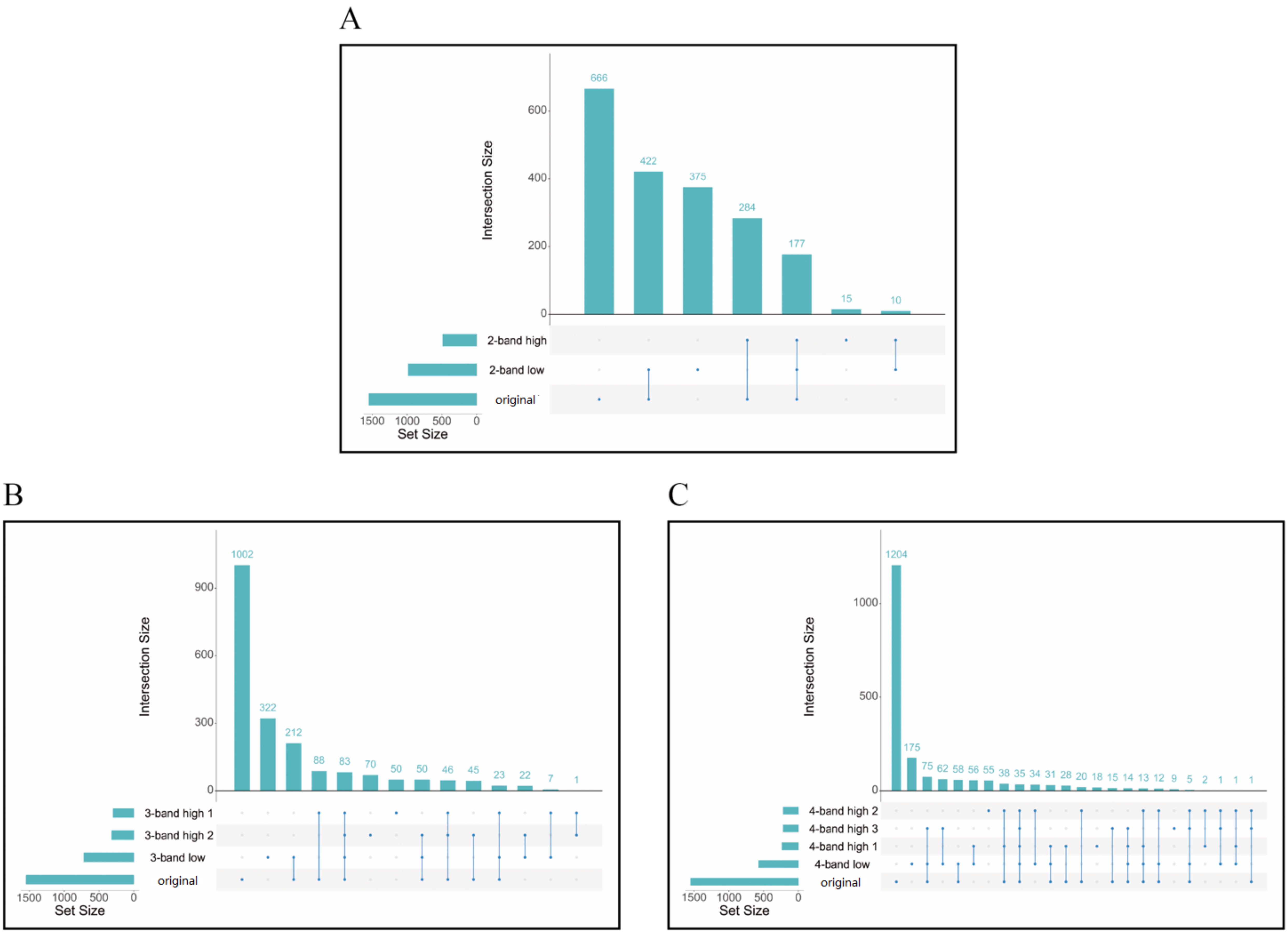

Fig M: The intersection of genes and cell-type related markers for CID4523. (A) The intersection of genes between canonical data and its different frequency components using 2-band DWT (Daub4). (B) Similar to (A), but using 3-band DWT. (C) Similar to (A), but using 4-band DWT.

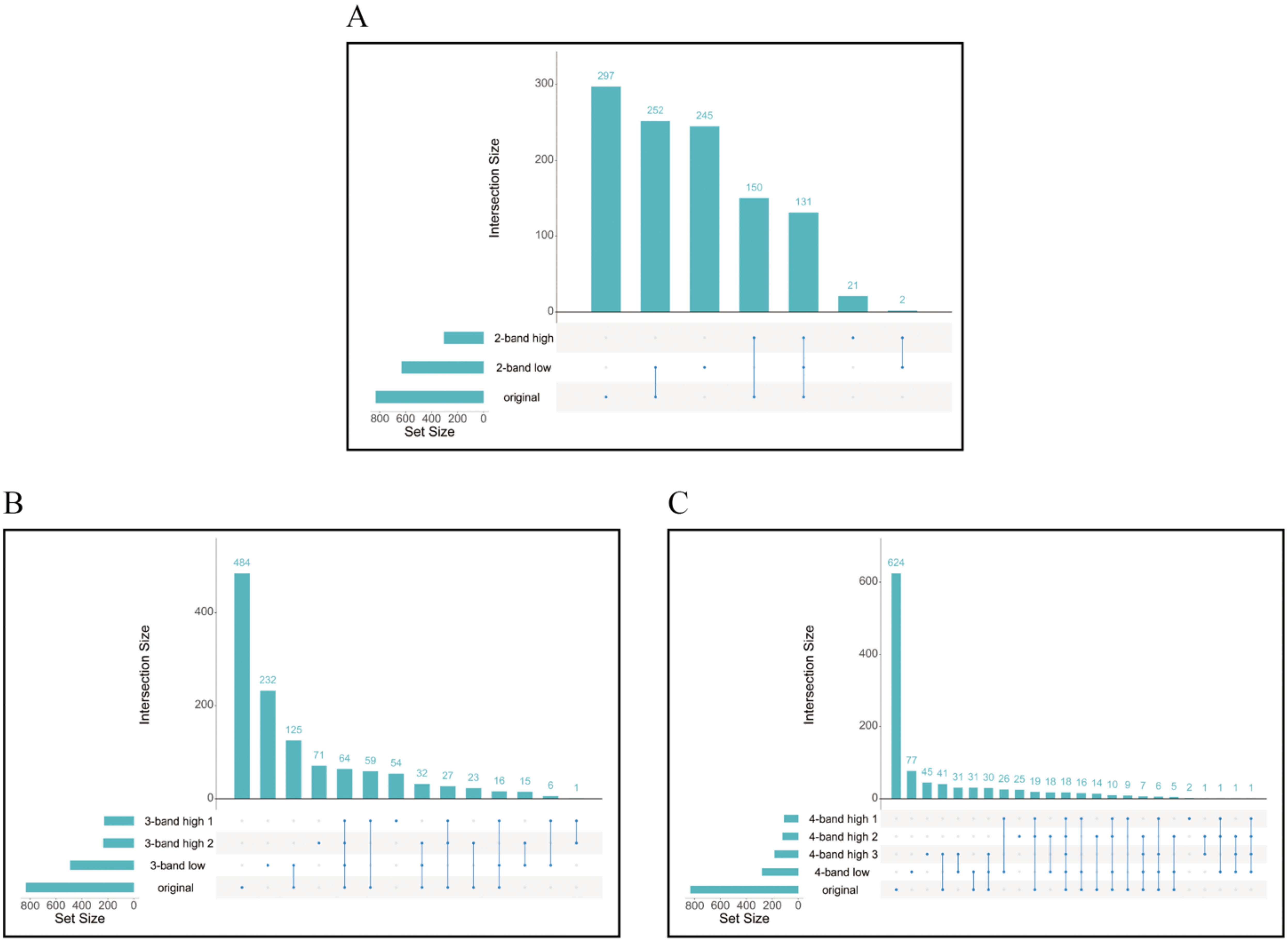

Fig N: The intersection of genes and cell-type related markers for PBMC dataset. (A) The intersection of genes between canonical data and its different frequency components using 2-band DWT (Daub4). (B) Similar to (A), but using 3-band DWT. (C) Similar to (A), but using 4-band DWT.

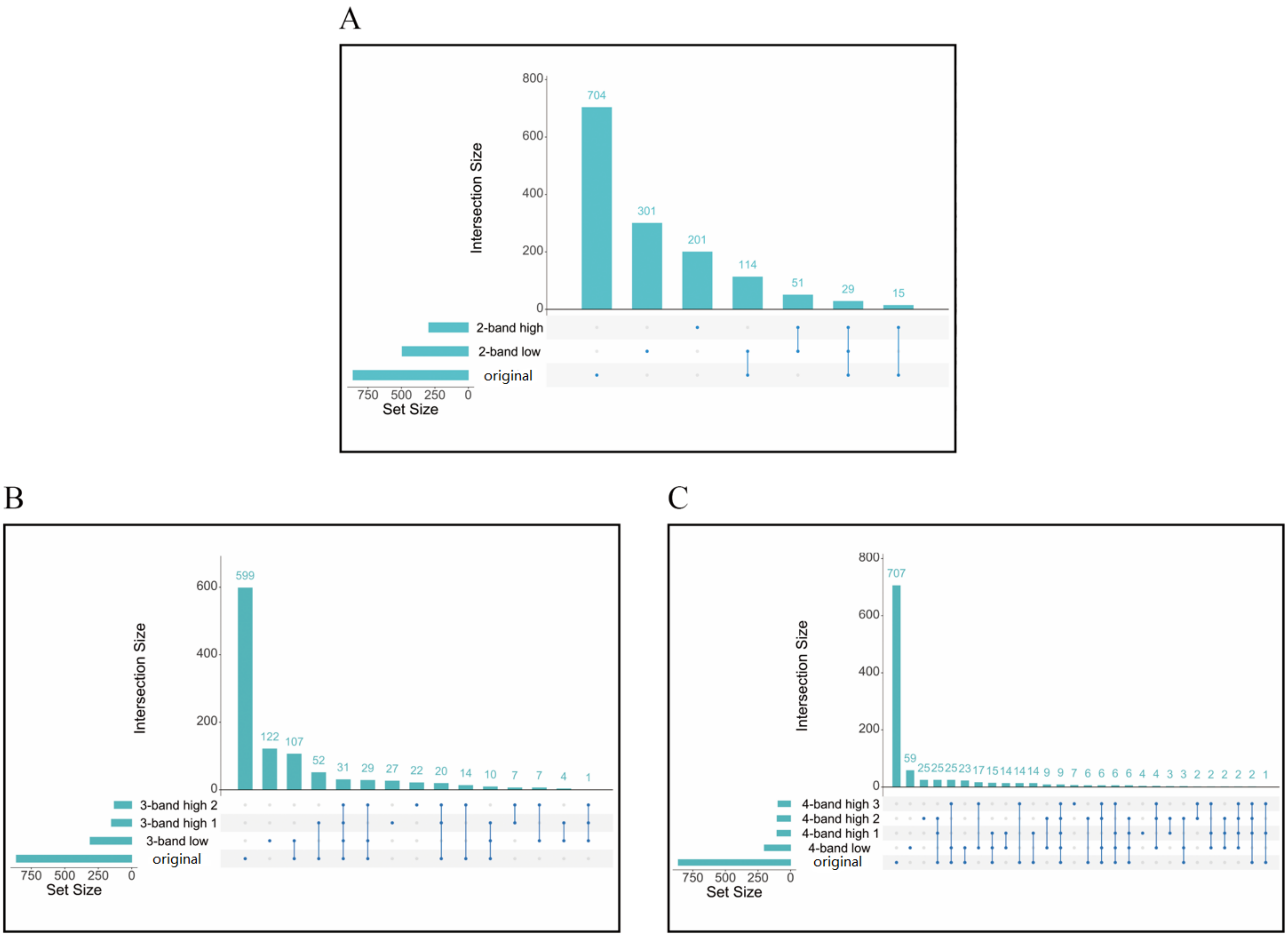

Fig O: The intersection of genes and cell-type related markers for colorectal cancer dataset . (A) The intersection of genes between canonical data and its different frequency components using 2-band DWT (Daub4). (B) Similar to (A), but using 3-band DWT. (C) Similar to (A), but using 4-band DWT.

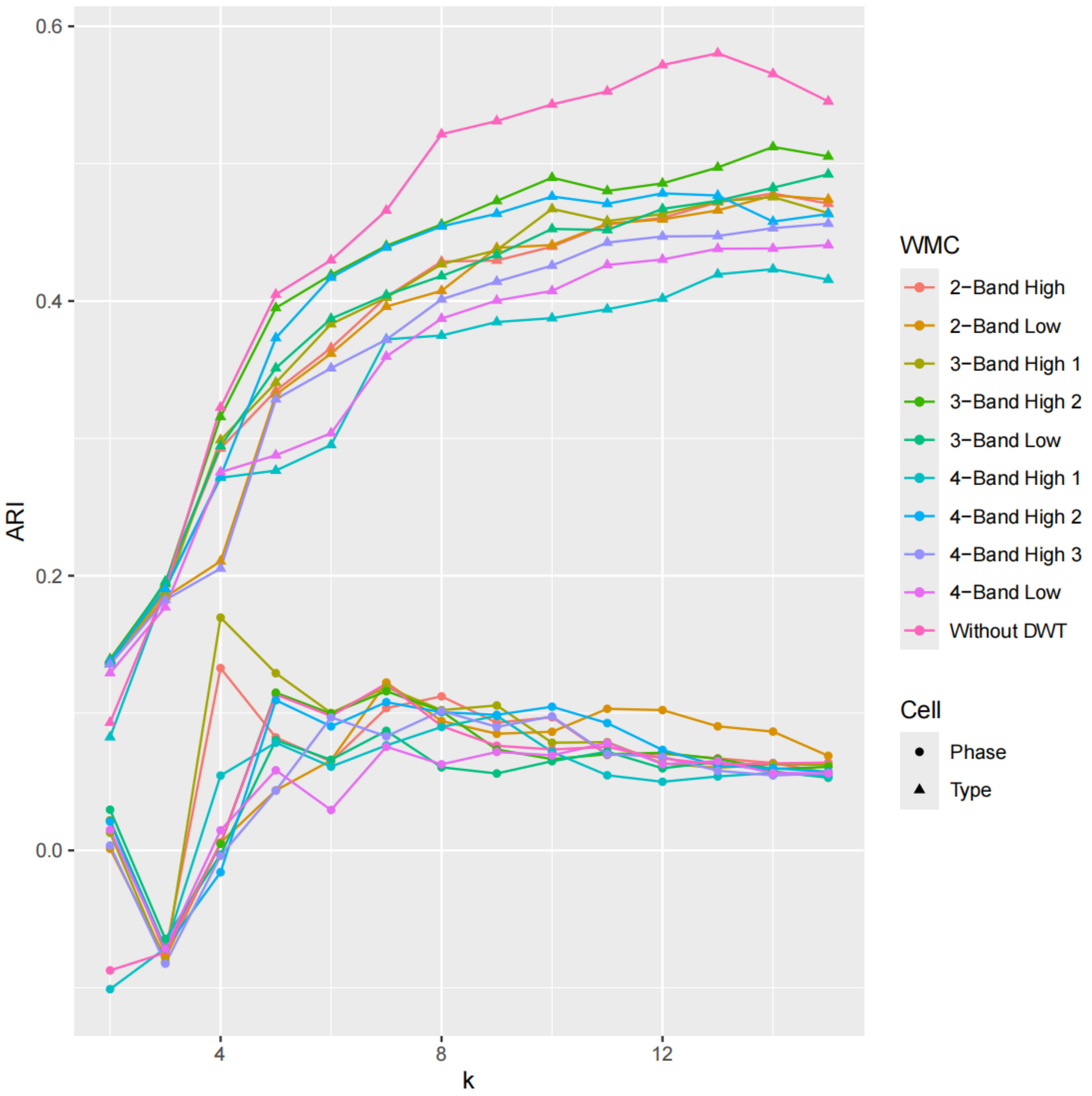

Fig P: ARI plots for ILC dataset with cell types and cell phases.

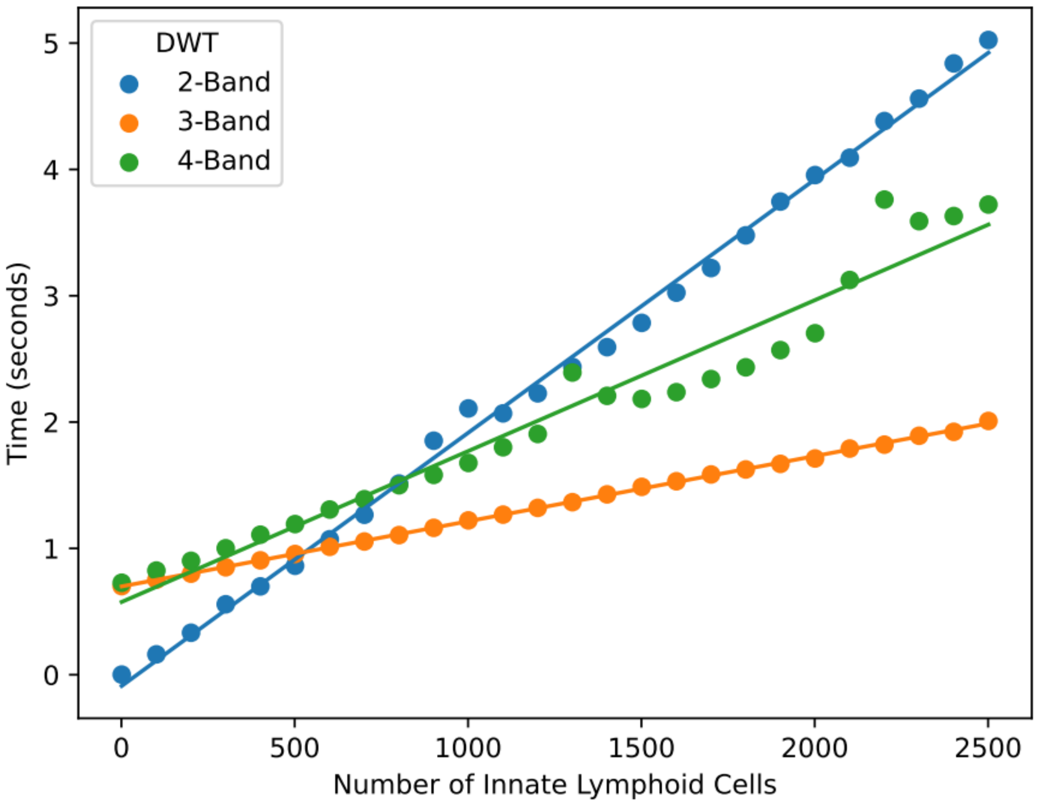

Fig Q: Running times for wavelet transform procedure with different sample sizes.

References

[S1] Ingrid Daubechies, "Ten Lectures on Wavelets", ISBN-13: 978-0-898712-74-2

[S2] P. Steffen; P.N. Heller; R.A. Gopinath; C.S. Burrus, "Theory of regular M-band wavelet bases', IEEE Transactions on Signal Processing, Volume: 41, Issue: 12, December 1993

[S3] Tony Lin, Shufang Xu, Qingyun Shi, Pengwei Hao, "An algebraic construction of orthonormal M-band wavelets with perfect reconstruction", Applied Mathematics and Computation Volume 172, Issue 2, 15 January 2006, Pages 717-730

[S4] Abdul Hasib Rahimyar, Hieu Quang Nguyen, Xiaodi Wang, "Stock Forecasting using M-Band Wavelet Based SVR and RNN-LSTMs Models" , the conference proceedings of 2019 2nd International Conference on Information Systems and Computer Aided Education (ICISCAE) by IEEE.
